# Dual‐Gene Edited Extracellular Vesicles Remodel the Redox Homeostasis to Inhibit Ferroptosis in Intervertebral Disc Degeneration

**DOI:** 10.1002/advs.77329

**Published:** 2026-08-20

**Authors:** Jing Yan, Yazhou Lin, Shuo Miao, Minghao Chao, Chengbai Dai, Ziwen Hao, Rui Chen, Ming Guan, Bin Pan, Fenglei Gao, Quan Zhou

**Affiliations:** ^1^ Department of Orthopedics The Affiliated Huai'an Hospital of Xuzhou Medical University Huaian China; ^2^ Department of Orthopedics Ruijin Hospital Shanghai Jiaotong University School of Medicine China; ^3^ Department of Orthopaedic Surgery Lishui Central Hospital and Fifth Affiliated Hospital of Wenzhou Medical University Zhejiang China; ^4^ Key Laboratory of New Drug Research and Clinical Pharmacy of Jiangsu Province Xuzhou Medical University Jiangsu China; ^5^ Department of Laboratory Medicine Huashan Hospital Shanghai Medical College Fudan University Shanghai China; ^6^ Department of Spine Surgery The Affiliated Hospital of Xuzhou Medical University Jiangsu China

**Keywords:** extracellular vesicles, ferroptosis, intervertebral disc degeneration, mFD‐PEVs

## Abstract

Intervertebral disc degeneration (IDD) is driven by ferroptosis of nucleus pulposus cells (NPCs) as a core pathological mechanism. Nucleus pulposus progenitor cells (NPPCs), exhibiting stem cell‐like properties, yield extracellular vesicles (PEVs) with high affinity for NPCs and enable targeted phenotypic regulation. However, natural PEVs possess limited bioactivity. Therefore, we constructed engineered mFD‐PEVs modified with FTH1 and DAB2. In vitro experiments demonstrated that mFD‐PEVs efficiently deliver FTH1, maintained SLC7A11/GPX4‐associated redox defense, suppress NPCs oxidative stress, and attenuate ferroptosis. Furthermore, in vivo studies confirmed the potent therapeutic efficacy of mFD‐PEVs in promoting intervertebral disc regeneration. Transcriptomic analysis further revealed that mFD‐PEVs predominantly modulate molecular pathways associated with ferroptosis and oxidative stress. In summary, the gene‐edited mFD‐PEVs reprogram redox homeostasis in NPCs, regulate ferroptosis, and promote intervertebral disc regeneration.

## Abstract 2

Dual‐engineered mFD‐PEVs couple DAB2‐mediated nucleus pulposus targeting with FTH1 mRNA delivery to restore iron/redox homeostasis in degenerative nucleus pulposus cells. By reinforcing SLC7A11/GPX4‐dependent antioxidant defense and limiting Fe^2^^+^ accumulation, mitochondrial dysfunction, ROS generation, and lipid peroxidation, mFD‐PEVs suppress ferroptosis, preserve disc structure, and promote intervertebral disc regeneration, offering a disease‐modifying extracellular‐vesicle strategy for IDD treatment.

## 1 Introduction

Low back pain, as a common degenerative spinal disorder, has become one of the major causes of disability worldwide [1]. Intervertebral disc degeneration (IDD) is the core pathological basis for low back pain [2, 3]. Dysfunction of nucleus pulposus cells (NPCs) has been proven to be a significant characteristic of the progression of IDD [4, 5]. Studies have shown that ferroptosis of NPCs is the main pathological mechanism of IDD. The SLC7A11/GPX4 signaling pathway, a key antioxidant axis in amino acid metabolism, plays a central role in regulating ferroptosis. SLC7A11 functions as a cysteine/glutamate antiporter within the Xc‐ system, maintaining intracellular glutathione (GSH) levels to activate GPX4, thereby enhancing resistance to lipid peroxidation [6, 7]. Concurrently, Fe^2+^ catalyzes the Fenton reaction, promoting ROS accumulation and intensifying oxidative stress [8, 9]. Ferritin heavy chain 1 (FTH1), a key iron homeostasis protein with ferroxidase activity, maintains iron metabolism balance by oxidizing and chelating Fe^2+^ [10, 11]. Downregulation of FTH1 exacerbates lipid peroxidation and ferroptosis, while its overexpression exerts protective effects by stabilizing iron metabolism [12, 13]. Although FTH1 downregulation is known to aggravate osteoarthritis [14, 15, 16], its role in IDD remains unclear. Our research results indicated significant FTH1 downregulation in degenerated disc tissues; Overexpression of FTH1 restores iron homeostasis, suppresses ferroptosis in NPCs, and correlates with the restoration of SLC7A11/GPX4 mediated redox defense. These findings thus provide a mechanistic rationale for treating IDD through the reestablishment of redox balance. Therefore, delivering FTH1 mRNA to NPCs represents an innovative therapeutic strategy.

While mRNA‐based gene therapy offers advantages such as strong targeting and high safety, improving transfection efficiency in NPCs remains a key clinical bottleneck [17, 18]. Vectors for therapeutic mRNA delivery also face safety and immunogenicity challenges [19, 20]. An ideal vector should enable efficient, targeted mRNA delivery without causing cellular or host damage or triggering immune responses [21, 22]. Extracellular vesicles (EVs), natural nanocarriers containing proteins and nucleic acids, offer inherent stability, low immunogenicity, and excellent tissue penetration [23, 24]. EVs can deliver mRNA that evades extracellular degradation and enables direct intracellular translation for transient protein expression [25, 26]. Surface modification of EVs can further enhance targeting specificity [27, 28]. Therefore, gene‐edited EVs possess therapeutic targeting capability and high efficacy. EVs derived from nucleus pulposus progenitor cells (NPPCs) (PEVs) regulate the phenotypic changes of nucleus pulposus cells [29]. NPPCs, originating from the embryonic notochord, possess stem cell‐like properties including self‐renewal and pluripotency, enabling them to maintain intervertebral disc functional integrity [30, 31]. Given their excellent regenerative potential, NPPCs have become an important cell source for intervertebral disc regeneration therapy [32, 33]. NPPCs resist various programmed cell deaths and are crucial for NPCs survival [34, 35]; their loss is a primary cause of NPCs dysfunction and IDD progression [36]. EVs are mainly taken up by target cells through pathways such as clathrin and caveolin [37]. DAB2, a clathrin‐associated protein involved in receptor endocytosis [38], has not been studied in IDD, but bioinformatics shows its significant downregulation in degenerated nucleus pulposus (NP) tissue. Notably, PEVs loaded with DAB2 significantly enhanced NPCs uptake. These findings suggest that degenerated NPCs may suffer dual impairments in antioxidant systems and endocytic function, further promoting IDD progression.

**SCHEME 1:**
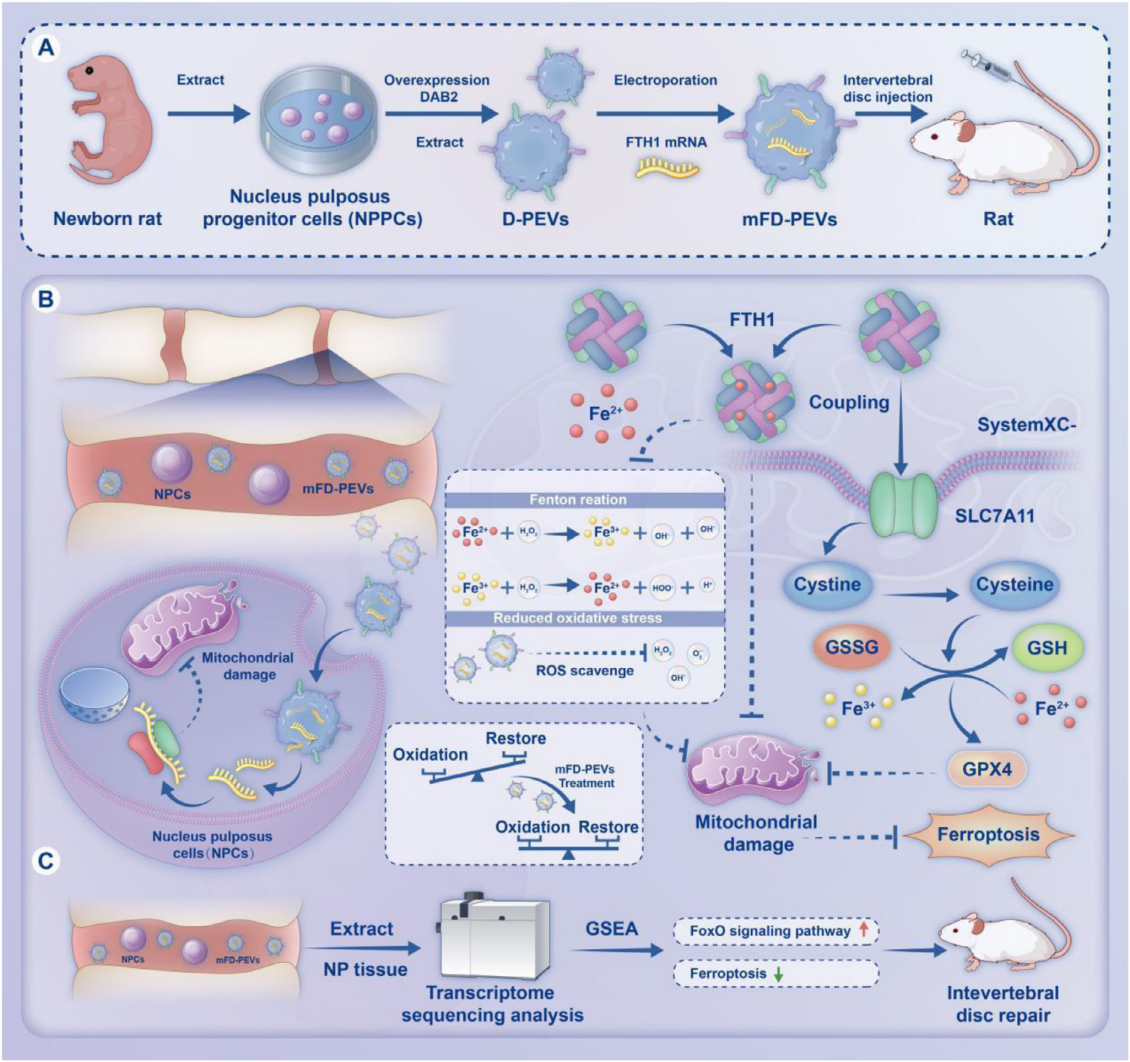
The preparation of mFD‐PEVs and the mechanism for regulating IDD. (A) Preparation of mFD‐PEVs. (B) mFD‐PEVs inhibit oxidative stress, maintain mitochondrial homeostasis, and suppress the ferroptosis mechanism in NPCs. (C) Transcriptomic analysis revealed that mFD‐PEVs predominantly modulate molecular pathways associated with ferroptosis and oxidative stress.

Based on these findings, we proposed a new strategy for treating IDD using genetically modified PEVs (Scheme 1). First, NPPCs were genetically edited with DAB2, from which engineered PEVs (D‐PEVs) were extracted to enhance targeting capability. Subsequently, FTH1 mRNA was loaded into D‐PEVs to construct mFD‐PEVs. This system can efficiently deliver FTH1 mRNA to degenerated NPCs, restore SLC7A11/GPX4‐associated redox defense, suppress oxidative stress in NPCs, attenuate ferroptosis, and thereby restore intervertebral disc height and delay IDD progression in vivo. Transcriptional sequencing analysis combined with in vitro experiments further indicated that mFD‐PEVs can restore the antioxidant capacity of cells and inhibit ferroptosis. This study not only revealed the protective mechanism of FTH1 in IDD, but also developed a targeted EVs delivery system, providing new tools and potential targets for the precise treatment of IDD.

## 2 Results and Discussion

### 2.1 IDD Involves Differences in the Expression of Ferroptosis and Endocytosis Pathways

**FIGURE 1:**
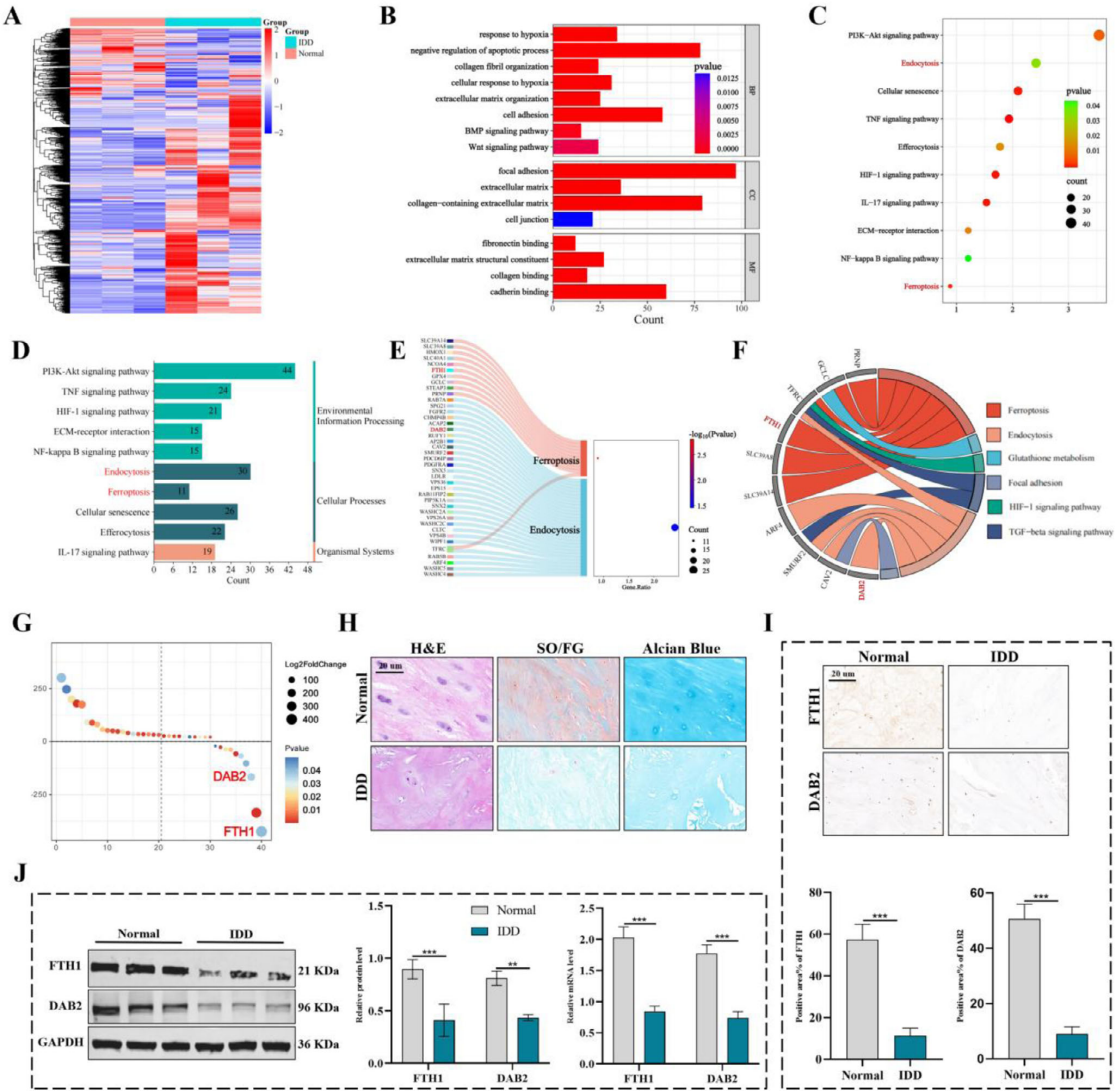
The expressions of FTH1 and DAB2 were significantly downregulated in degenerated NP tissues. (A) Visualization processing of differentially expressed genes using heat maps. (B) GO was used to conduct functional enrichment analysis on differentially expressed genes. (C) KEGG demonstrates differential pathways enriched by differentially expressed genes, including ferroptosis and endocytosis pathways. (D) Ferroptosis and endocytosis pathways are involved in cellular processes. (E, F) Genes involved in ferroptosis and endocytosis pathways. (G) Expression results of all genes in ferroptosis and endocytosis pathways. (H) Verification of clinical NP tissue morphology by HE, Safranin O‐Fast Green and alcian blue. *n* = 3. (I) Immunohistochemical verification of FTH1 and DAB2 in NP tissue. *n* = 3, ^***^ *p* < 0.001. (J) Protein and gene expression of FTH1 and DAB2 in NP tissue. *n* = 3, ^**^ *p* < 0.01; ^***^ *p* < 0.001. Data are expressed as mean ± standard deviation. Significance was determined using a two‐tailed Student's *t*‐test.

In recent years, bioinformatics analysis of microarray data has been widely used to identify key genes related to diseases and for subsequent analysis. Using IDD as a model, gene chip technology enables comprehensive detection of expression changes in genes implicated in IVDD pathogenesis, thereby allowing systematic screening of pivotal genes critically involved in disease initiation and progression [39]. Research has confirmed that ferroptosis is of great significance in degenerative diseases such as IDD, osteoarthritis, and liver cell degeneration [40]. The pathological process of IDD involves many links and pathways. Therefore, we screened the transcriptome data of the NP tissue to analyze the important pathological processes in IDD. We conducted a heat map and volcano map visualization analysis of the mRNA transcriptome dataset through bioinformatics (Figure 1A; Figure S1A). Subsequently, functional enrichment analyses were conducted on all differentially expressed genes (Figure 1B, C). It is worth noting that ferroptosis and endocytosis pathways show expression differences in IDD (Figure 1C), and both are related to cellular processes (Figure 1D). The heat map clearly lists the expression trends of all genes in ferroptosis and endocytosis pathways (Figure S1B,C). Subsequently, we presented some downregulated genes included in ferroptosis and endocytosis pathways (Figure 1E, F). To screen the most significant genes in the ferroptosis and endocytosis pathways, we visualized the expression of all genes in the two pathways. The results showed that FTH1 and DAB2 were the genes with the most significant downregulation (Figure 1G), and also the hub genes (Figure S1D). Figure S1E,F indicate that FTH1 and DAB2 respectively play roles in ferroptosis and endocytosis pathways. From this, we speculate that during the process of IDD, the antioxidant system and endocytic function are impaired and unable to self‐repair, thereby promoting the progression of IDD. Therefore, we selected FTH1 and DAB2 as the research targets. Subsequently, we collected the expression of FTH1 and DAB2 in clinical NP tissues for verification. HE and Safranin O‐Fast Green staining indicated that, compared with normal NP tissue, the collagen fibers in degenerated NP tissue were disordered, the number of cells was reduced, and alcian blue staining showed that the contents of proteoglycan and collagen in degenerated NP tissue were decreased (Figure 1H). The immunohistochemical results indicated that FTH1 and DAB2 were downregulated in the degenerated NP tissue (Figure 1I). Moreover, the results of Western blot (WB) and RT‐PCR indicated that the expressions of FTH1 and DAB2 were significantly downregulated in degenerated NP tissues (Figure 1J). These results indicate that the downregulation of FTH1 and DAB2 expression has significant research significance in the process of IDD.

### 2.2 TBHP Induces Oxidative Stress in NPCs and Inhibits the Expression of FTH1 and DAB2

**FIGURE 2:**
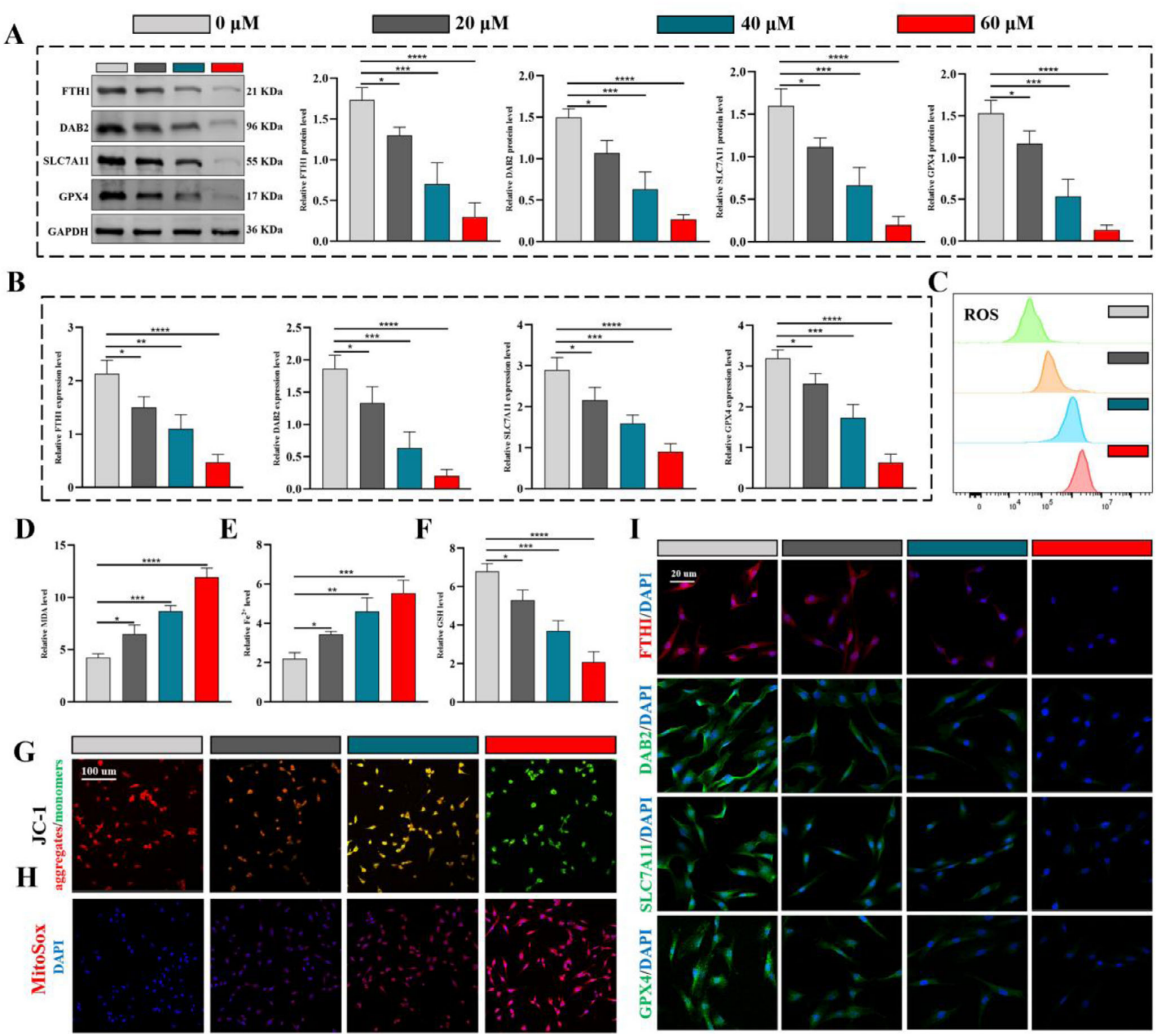
TBHP inhibits the expression of FTH1 and DAB2, inducing ferroptosis in NPCs. (A, B) The protein expressions of FTH1, DAB2, SLC7A11 and GPX4 were detected by WB and RT‐PCR. *n* = 3, ^*^ *p* < 0.01; ^**^ *p* < 0.01; ^***^ *p* < 0.001; ^****^ *p* < 0.0001. (C) The content of ROS induced by different concentrations of TBHP was detected by flow cytometry. *n* = 3. (D) MDA content. *n* = 3, ^*^ *p* < 0.01; ^***^ *p* < 0.001; ^****^ *p* < 0.0001. (E) Fe^2+^ ion content. *n* = 3, ^*^ *p* < 0.01; ^**^ *p* < 0.01; ^****^ *p* < 0.0001. (F) Consumption of GSH. *n* = 3, ^*^ *p* < 0.01; ^***^ *p* < 0.001; ^****^ *p* < 0.0001. (G) JC‐1 was used to detect changes in mitochondrial membrane potential. *n* = 3. (H) MitoSox detects the content of superoxides in mitochondria. *n* = 3. (I) Cellular immunofluorescence was used to detect protein expression. *n* = 3. Data are expressed as mean ± standard deviation. Significance was determined using one‐way ANOVA.

It is noteworthy that oxidative stress plays a pivotal role in ferroptosis. Characterized by excessive production of ROS, oxidative stress serves as the primary driver of ferroptosis by disrupting cellular redox homeostasis and impairing key antioxidant enzymes. This pro‐oxidative environment facilitates the accumulation of toxic lipid peroxides, thereby exacerbating cell death [41]. TBHP is widely used to induce oxidative stress models in NPCs and promote mitochondrial dysfunction [42]. When different concentrations of TBHP were co‐cultured with NPCs for 24 h, we found that the injury indicators of NPCs were concentration‐dependent. With the increase of TBHP concentration, the protein and mRNA levels of FTH1 and DAB2 were downregulated to varying degrees (Figure 2A, B). Moreover, SLC7A11 and GPX4 protein and mRNA levels were also downregulated in a concentration‐dependent manner (Figure 2A, B). In addition, we conducted a series of assays to evaluate cellular antioxidant capacity. Flow cytometry results indicated that with the increase of TBHP concentration, the intracellular ROS content significantly increased (Figure 2C). The contents of malondialdehyde (MDA) and Fe^2+^ showed concentration‐dependent increases (Figures 2D,E). Meanwhile, TBHP promotes the consumption of GSH (Figure 2F). Lipid peroxidation assay results also showed that cellular oxidative levels gradually increased with rising TBHP concentrations (Figure S2A). Meanwhile, TBHP inhibited cell proliferation in a concentration‐dependent manner (Figure S2B). JC‐1 and MitoSox respectively reflect the changes in mitochondrial membrane potential and superoxide content. The results of JC‐1 and MitoSox indicated that with the increase of TBHP concentration, the mitochondrial membrane potential of cells gradually decreased, and the content of superoxide in mitochondria increased, suggesting mitochondrial damage (Figures 2G,H). Then, the results of cellular immunofluorescence proteins indicated that the protein expressions of FTH1, DAB2, SLC7A11 and GPX4 were concentration‐dependent downregulated (Figure 2I). The above results indicate that TBHP damages the redox balance and inhibits vitality of NPCs. To determine whether TBHP indeed induces ferroptosis in NPCs, we conducted the following experiments. TBHP significantly suppressed the protein expression of SLC7A11 and GPX4, whereas the ferroptosis inhibitor Fer‐1 reversed this trend (Figure S2C). The downregulation of SLC7A11 and GPX4 by TBHP was also observed at the gene expression level, and Fer‐1 effectively restored their expression (Figure S2D, E). Furthermore, the characteristic molecular markers of ferroptosis were validated: TBHP significantly increased MDA and Fe^2^^+^ levels while decreasing GSH levels; Fer‐1 maintained the stability of these molecular levels (Figure S2F–H). Concurrently, Fer‐1 markedly suppressed lipid peroxidation levels (Figure S2I) and reduced intracellular ROS levels (Figure S2J) in the TBHP‐induced group. Finally, Fer‐1 reversed the TBHP‐induced decline in cell viability (Figure S2K). In conclusion, TBHP indeed induces ferroptosis in NPCs. Therefore, in the subsequent experiments, we chose 60 µm concentration of TBHP to prepare the NPCs injury model. These results indicate that TBHP impairs the antioxidant capacity of cells, promotes mitochondrial dysfunction, and inhibits the expression of FTH1 and DAB2, ultimately inducing ferroptosis in NPCs. Subsequently, we further verified the correlation between the TBHP‐induced NPCs model and the molecular characteristics of clinical IDD. Compared with the normal group, the IDD group exhibited downregulated protein and gene levels of SLC7A11 and GPX4 (Figure S3A). Similarly, relative to the control group, the TBHP‐treated group showed decreased expression of FTH1, DAB2, SLC7A11, and GPX4 at both protein and gene levels (Figure S3B, C). Moreover, a summary heatmap of the gene expression profiles for FTH1, DAB2, SLC7A11 and GPX4 revealed similar expression patterns between the TBHP‐induced model and clinical IDD specimens (Figure S3D). These results indicate that the TBHP‐induced NPCs model at least recapitulates the core redox‐related molecular features of clinical IDD.

### 2.3 FTH1 Inhibits Oxidative Stress in NPCs and Maintains Mitochondrial Homeostasis

**FIGURE 3:**
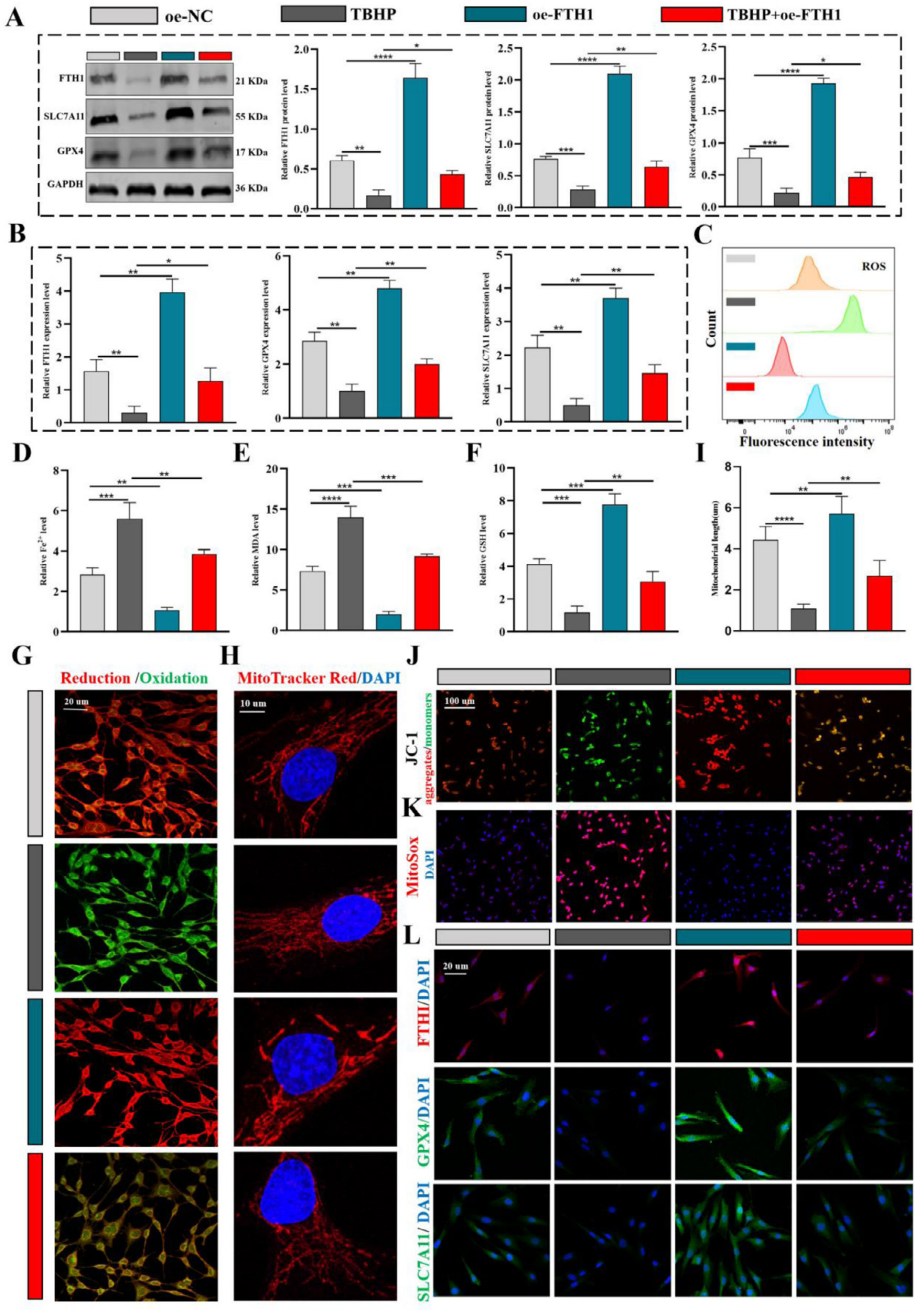
FTH1 inhibits oxidative stress in NPCs and maintains mitochondrial homeostasis. (A, B) The expressions of FTH1, SLC7A11 and GPX4 were detected by WB and RT‐PCR. *n* = 3, ^*^ *p* < 0.01; ^**^ *p* < 0.01; ^***^ *p* < 0.001; ^****^ *p* < 0.0001. (C) Detection of ROS content by flow cytometry. *n* = 3. (D) Iron ion content. *n* = 3, ^**^ *p* < 0.01; ^***^ *p* < 0.001. (E) MDA content. *n* = 3, ^***^ *p* < 0.001; ^****^ *p* < 0.0001. (F) Consumption of GSH. *n* = 3, ^**^ *p* < 0.01; ^***^ *p* < 0.001; ^****^ *p* < 0.0001. (G) FTH1 attenuates lipid peroxidation levels in the TBHP‐induced group. *n* = 3. (H, I) MitoTracker Red was used to assess mitochondrial morphology. *n* = 3, ^**^ *p* < 0.01; ^****^ *p* < 0.0001. (J) JC‐1 detects mitochondrial membrane potential of cells. *n* = 3. (K) MitoSox detects the content of superoxides in mitochondria. *n* = 3. (L) Cellular immunofluorescence was used to detect protein expression. *n* = 3. Data are expressed as mean ± standard deviation. Significance was assessed using one‐way ANOVA followed by Tukey's multiple‐comparison test.

This part of the experiment further verifies the regulatory effect of FTH1 on ferroptosis in NPCs. We co‐cultured NPCs with TBHP and FTH1 plasmids. The results of WB and RT‐PCR indicated that FTH1 significantly reversed the inhibitory effect of TBHP on the expression of FTH1, SLC7A11 and GPX4 (Figure 3A,B). We have confirmed that TBHP promotes the accumulation of ROS, the increase of iron ion content and the elevation of MDA content, accelerates the consumption of GSH, and ultimately induces oxidative stress in NPCs. Flow cytometry results indicated that FTH1 significantly inhibited ROS accumulation, reduced iron ion and MDA content, increased GSH content, and weakened the damage of TBHP to cells (Figure 3C–F). TBHP induces cellular lipid oxidation, whereas FTH1 significantly maintains the redox homeostasis of lipids (Figure 3G). Subsequently, we examined the morphology and function of mitochondria. The MitoTracker Red results confirmed that TBHP caused mitochondria to become granular, indicating that the mitochondrial structure was damaged. Moreover, FTH1 significantly restored mitochondrial length and improved mitochondrial morphology (Figure 3H,I). On the other hand, compared with the TBHP group, FTH1 could restore mitochondrial membrane potential stability and reduce mitochondrial superoxide content (Figure 3J,K). The results of cellular immunofluorescence indicated that FTH1 significantly recovered the expression of FTH1, SLC7A11 and GPX4 (Figure 3L). Overall, FTH1 inhibits oxidative stress damage in cells and maintains mitochondrial homeostasis.

Research found that knocking down the expression of FTH1 significantly induced joint cartilage damage, promoted ferroptosis of chondrocytes, and aggravated osteoarthritis [43]. Studies have confirmed that FTH1 can maintain iron ion homeostasis and prevent ferroptosis, and overexpression of FTH1 inhibits the production of peroxides, reduces the consumption of GSH and inhibits the inactivation of GPX4, and inhibits osteoarthritis [44]. Currently, there are still insufficient reports on the role of FTH1 in IDD. In summary, we have confirmed that compared to control nucleus pulposus (NP) tissue, FTH1 is expressed at a lower level in IDD. Overexpression of FTH1 significantly inhibits ferroptosis of NPCs induced by TBHP and maintains mitochondrial homeostasis. The results indicate that FTH1 is of great significance in regulating the homeostasis of NPCs. However, the method of efficient targeting of FTH1 plasmids to nucleus pulposus cells is still scarce.

### 2.4 Preparation and Characterization of mFD‐PEVs

**FIGURE 4:**
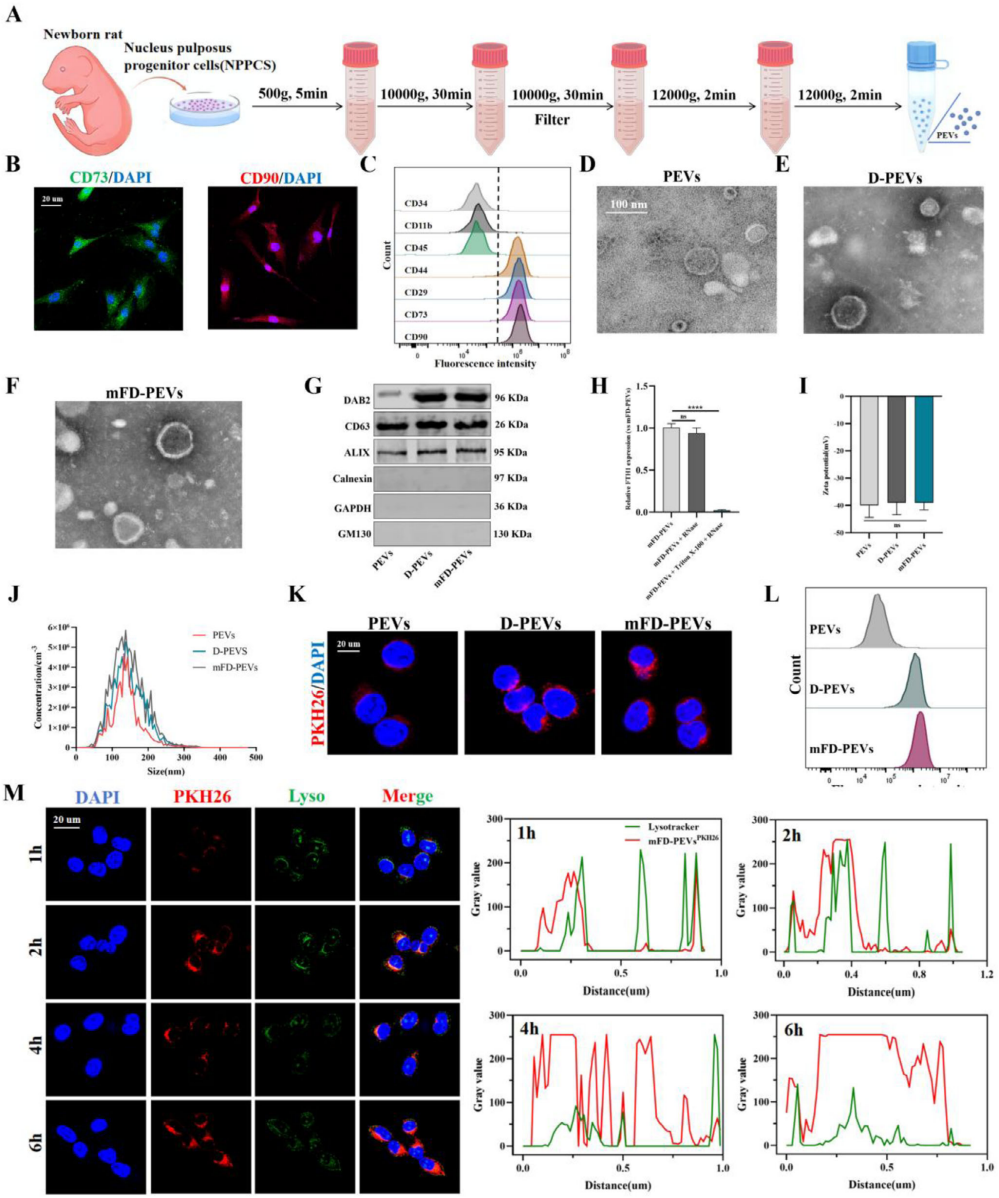
Preparation and Characterization of mFD‐PEVs. (A) The method for extracting PEVs. (B) Verification of NPPCs marker proteins by cellular immunofluorescence. *n* = 3. (C) Flow cytometry was used to analyze the characteristic features of NPPCs. *n* = 3. (D, E, F) TEM images of PEVs and D‐PEVS. *n* = 3. (G) Validation of PEVs marker proteins. *n* = 3. (H) FTH1 mRNA is localized within the lumen of mFD‐PEVs. *n* = 3, ^****^ *p* < 0.0001; ns, not significant. (I) The Zeta potential of PEVs, D‐PEVs, and mFD‐PEVs. *n* = 3, ns, not significant. (J) NTA of particle size. *n* = 3. (K, L) The uptake of PEVs by NPCs. *n* = 3. (M) Lysosomal escape. *n* = 3. Data are expressed as mean ± standard deviation. Significance was determined using one‐way ANOVA.

We extracted NPPCs from neonatal rats and then analyzed the related characteristics of NPPCs through immunofluorescence staining and flow cytometry. The results showed that NPPCs highly expressed stem cell markers CD44, CD29, CD90 and CD73, while the expression levels of CD11b, CD45 and CD34 were significantly decreased, confirming that it had progenitor cell characteristics (Figure 4B,C). Figure 4A shows the method for extracting PEVs. The results of TEM showed that PEVs had a typical vesicle structure (Figure 4D). To verify the uptake preference of NPCs for EVs from different sources, we simultaneously prepared MSCs‐EVs and verified their characterization through transmission electron microscopy (TEM), WB and nanoparticle tracking analysis (NTA) (Figure S4A,B). PEVs and MSCs‐EVs were labeled with PKH26 fluorescent dye and then co‐cultured with NPPCs respectively. Confocal microscopy and flow cytometry analysis indicated that the uptake efficiency of PEVs by NPPCs was significantly higher than that by MSCs‐EVs (Figure S4C, D). Based on the above results, we chose PEVs as the gene delivery vector. DAB2‐enriched engineered PEVs (D‐PEVs) were prepared by transfecting NPPCs with a DAB2‐overexpression plasmid (Figure 4E). Subsequently, FTH1 was introduced into D‐PEVs by electroporation technology, and the double‐modified engineered PEVs (mFD‐PEVs) were constructed (Figure 4F). WB analysis revealed that PEVs, D‐PEVs, and mFD‐PEVs retained the EVs‐associated markers ALIX and CD63. In contrast, the non‐EVs contaminant markers Calnexin, GM130, and GAPDH were undetectable. Additionally, DAB2 was prominently expressed at the protein level in mFD‐PEVs (Figure 4G). Collectively, these data indicate that mFD‐PEVs exhibit high purity and substantial DAB2 loading (Figure 4G and Figure S5A). Protease K cannot penetrate the intact lipid bilayer and therefore only digests membrane proteins exposed on the outer surface of EVs. In contrast, Triton X‐100 promotes membrane permeabilization, allowing protease K to degrade all intravesicular proteins [45]. Thus, treating mFD‐PEVs with protease K combined with Triton X‐100 enables us to determine whether DAB2 is localized on the vesicle surface or within the lumen, thereby revealing the mechanism underlying its pro‐endocytic activity. WB analysis revealed that, compared with the mFD‐PEVs group, proteinase K markedly digested DAB2, whereas the addition of Triton X‐100 did not alter the effect of proteinase K on DAB2 protein expression. These findings indicate that DAB2 is predominantly localized on the surface of mFD‐PEVs (Figure S5B, C). Therefore, we concluded that DAB2 regulates the uptake process by altering the surface composition of mFD‐PEVs. Subsequently, we analyzed the localization of FTH1 mRNA in mFD‐PEVs. Compared with untreated mFD‐PEVs, treatment with RNase, which has the ability to degrade RNA, alone did not cause significant degradation of FTH1 mRNA. However, upon combined treatment with Triton X‐100, FTH1 mRNA was markedly degraded, indicating that the FTH1 mRNA is localized within the lumen of mFD‐PEVs (Figure 4H). Restriction endonuclease digestion confirmed successful FTH1 mRNA loading into mFD‐PEVs (Figure S5D). Electroporation efficiency analysis revealed that approximately 40% FTH1 mRNA loading efficiency (Figure S5E). The Zeta potential assay showed that there were no significant differences in Zeta potential among the PEVs, D‐PEVs, and mFD‐PEVs (Figure 4I). Nanoparticle tracking analysis (NTA) revealed that the mean diameters of PEVs, D‐PEVs, and mFD‐PEVs were 140.61 ± 42.03, 145.98 ± 44.64, and 145.51 ± 46.34 nm, respectively (Figure 4J). Collectively, these characterization results demonstrate that DAB2 is highly loaded on the surface of mFD‐PEVs, whereas FTH1 mRNA is encapsulated within the vesicles. Furthermore, electroporation did not compromise the stability of mFD‐PEVs. Functional verification experiments demonstrated that, compared with common PEVs, DAB2 modification significantly enhanced the uptake efficiency of PEVs by NPCs (Figure 4K,L). Subsequently, NPCs were cultured with different endocytic inhibitors. The endocytic process of mFD‐PEVs labeled with PKH26 was observed. Among them, Chlorpromazine: an inhibitor used to block the endocytosis mediated by the membrane; Wortmannin: an inhibitor of phosphatidylinositol 3‐kinase; Dynasore: a dynamin inhibitor; Filipin: an inhibitor related to the endocytosis dependent on small vesicles [46]. The results of laser confocal microscopy and flow cytometry showed that compared with the control group, the Chlorpromazine group significantly inhibited the uptake of mFD‐PEVs, while the other groups showed no significant changes. This indicates that mFD‐PEVs enter the cells through endocytosis mediated by clathrin (Figure S6A,B). Live/dead cell staining demonstrated that PEVs, D‐PEVs, and mFD‐PEVs exhibited no cytotoxicity (Figure S6C). Lysosomal escape is a key link for nanoparticles to achieve intracellular functions [47]. By dynamically tracking the distribution of PKH26‐labeled mFD‐PEVs in NPCs through confocal microscopy, we found that at 2 h of co‐culture, the fluorescence signal of PKH26 showed complete co‐localization with the lysosomal marker. After 4 h, some fluorescence signals showed spatial separation. By 6 h, only a small amount of PKH26 fluorescence overlapped with lysosomes (Figure 4M). This spatiotemporal dynamic change confirms that mFD‐PEVs can achieve intracellular delivery through the endosome‐lysosome escape pathway. Flow cytometry was subsequently employed to assess the uptake of mFD‐PEVs by NPCs induced by TBHP at various time points. The results demonstrated that NPCs uptake of mFD‐PEVs increased progressively over time, exhibiting a time‐dependent manner (Figure S7A). Concurrently, internalization of mFD‐PEVs into NPCs leads to time‐dependent increases in FTH1 mRNA and protein expression (Figure S7B–E). Collectively, these results confirm that mFD‐PEVs successfully deliver FTH1 mRNA into NPCs and support its subsequent translation.

### 2.5 mFD‐PEVs Stabilize Mitochondrial Homeostasis in NPCs and Suppress Ferroptosis

**FIGURE 5:**
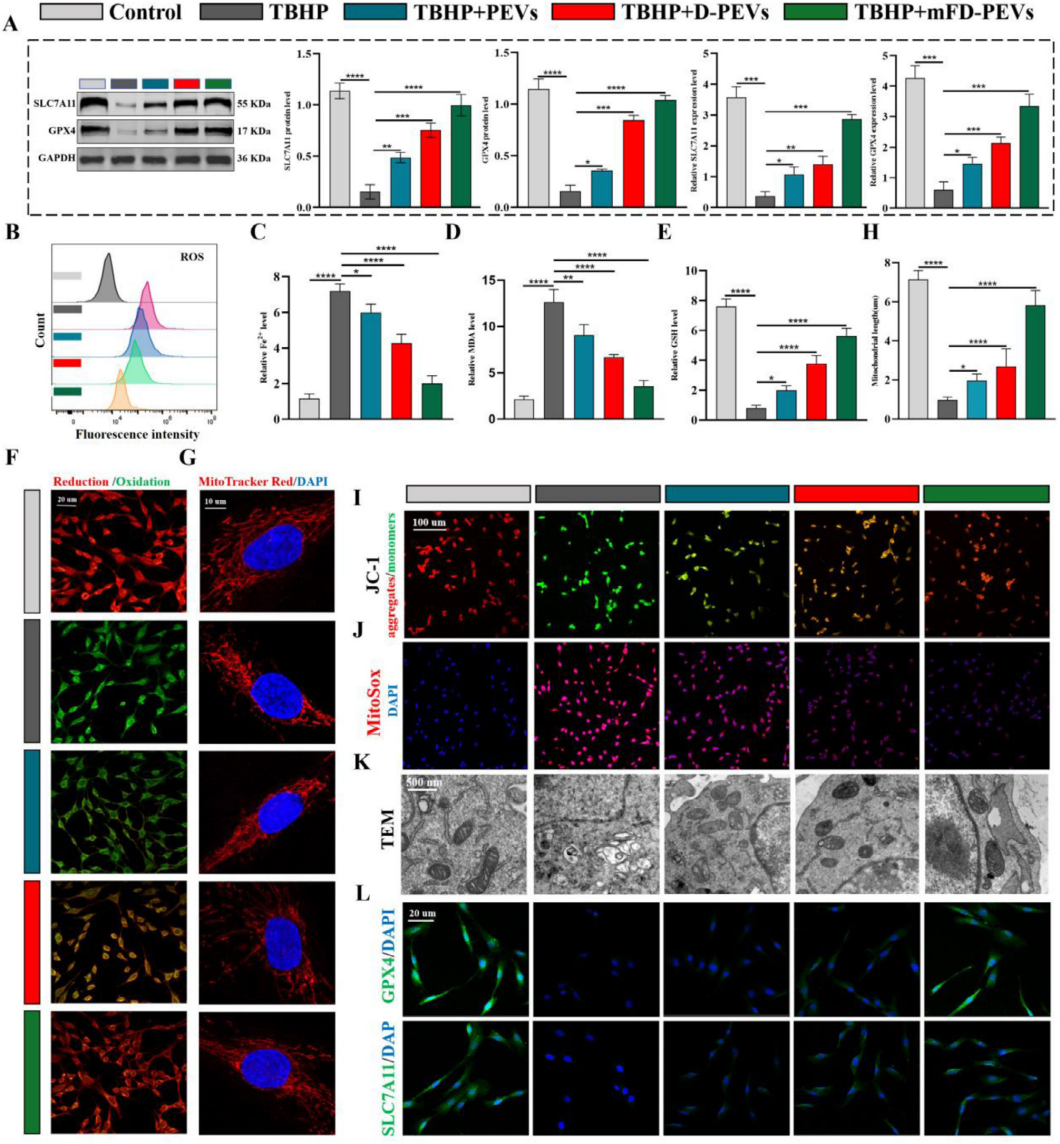
mFD‐PEVs inhibit ferroptosis in NPCs. (A) The expressions of SLC7A11 and GPX4 were detected by WB and RT‐PCR. *n* = 3, ^*^ *p* < 0.01; ^**^ *p* < 0.01; ^***^ *p* < 0.001; ^****^ *p* < 0.0001. (B) Changes in ROS content. *n* = 3. (C) Fe^2+^ ion content. *n* = 3, ^*^ *p* < 0.01; ^****^ *p* < 0.0001. (D) MDA content. *n* = 3, ^**^ *p* < 0.01; ^****^ *p* < 0.0001. (E) Consumption of GSH. *n* = 3, ^*^ *p* < 0.01; ^****^ *p* < 0.0001. (F) lipid peroxidation levels. *n* = 3. (G, H) MitoTracker Red detects changes in mitochondrial length. *n* = 3, ^*^ *p* < 0.01; ^****^ *p* < 0.0001. (I) JC‐1 detects changes in mitochondrial membrane potential. *n* = 3. (J) MitoSox detects the content of superoxides in mitochondria. *n* = 3. (K) TEM imaging of mitochondrial morphology. *n* = 3. (L) Cellular immunofluorescence was used to detect the expression of SLC7A11 and GPX4. *n* = 3. Data are expressed as mean ± standard deviation. Significance was determined using one‐way ANOVA.

The results of WB and RT‐PCR indicated that PEVs significantly restored the expression of SLC7A11 and GPX4, and the effect of mFD‐PEVs in promoting protein expression was superior to that of other treatment groups (Figure 5A). mFD‐PEVs have greater advantages in inhibiting ROS accumulation, reducing Fe^2+^ ion and MDA content, and increasing GSH content (Figure 5B–E). Excessive Fe^2+^ catalyzes the generation of a large amount of ROS through the Fenton reaction, thereby intensifying lipid peroxidation [48]. TBHP induces cellular lipid oxidation, whereas mFD‐PEVs significantly maintained lipid redox homeostasis (Figure 5F). On the one hand, mFD‐PEVs significantly restored mitochondrial length and improved mitochondrial morphology (Figure 5G,H). On the other hand, compared with other groups, mFD‐PEVs demonstrated superior performance in restoring mitochondrial membrane potential stability and reducing mitochondrial superoxide content (Figure 5I,J). During ferroptosis, mitochondria undergo characteristic morphological alterations, including volume contraction, increased membrane density, reduction or complete loss of cristae, and outer membrane rupture [49]. Therefore, we took TEM images of mitochondria. TBHP induces mitochondrial crest rupture and vacuole formation. PEVs, D‐PEVs and mFD‐PEVs all restore the structure of mitochondria to varying degrees, among which the restoration effect of mFD‐PEVs is the closest to the normal mitochondrial structure (Figure 5K). The results of cellular immunofluorescence indicated that mFD‐PEVs had the most significant effect in restoring the expression of FTH1, SLC7A11 and GPX4 among all treatment groups (Figure 5L). Erastin triggers ferroptosis by blocking the function of the SLC7A11 protein in the System Xc^−^and impairing intracellular antioxidant defenses [50]. Therefore, erastin was used to determine whether inhibition of the SLC7A11/GPX4 axis could compromise the protective effects of mFD‐PEVs. Compared with the control group, mFD‐PEVs significantly restored the protein and gene expression levels of SLC7A11 and GPX4, whereas erastin markedly reversed this effect (Figure S8A, B). Cellular immunofluorescence results further confirmed that erastin suppressed the protein expression of SLC7A11 and GPX4 in the mFD‐PEVs group (Figure S8C). To further determine whether FTH1 is functionally indispensable for NPCs in counteracting ferroptosis, we employed si‐FTH1 to interfere with the therapeutic effects of mFD‐PEVs on NPCs. Compared with the TBHP group, mFD‐PEVs treatment restored FTH1 and DAB2 expression, maintained SLC7A11 and GPX4 levels (Figure S9A, B), increased GSH content (Figure S9C), and suppressed Fe^2+^ accumulation (Figure S9D), MDA levels (Figure S9E), ROS production (Figure S9F), and lipid peroxidation (Figure S9G). However, these protective effects were markedly attenuated upon si‐FTH1 transfection, indicating that FTH1 serves as a critical functional component of the mFD‐PEVs‐mediated protection. This also suggested that FTH1 may influence the endogenous expression of DAB2 in NPCs. To validate this hypothesis, we examined the regulation of DAB2 expression by PEVs, D‐PEVs, and mFD‐PEVs using WB and RT‐qPCR. Relative to the TBHP group, all three PEVs types significantly restored both protein and mRNA levels of DAB2, with mFD‐PEVs exhibiting the most prominent restorative effect. This indicated that mFD‐PEVs, while achieving FTH1 delivery and translation, concurrently promote endogenous DAB2 expression (Figure S10A–C). Nevertheless, the precise relationship between FTH1 and DAB2 requires further investigation. Compared with the control group, si‑FTH1 markedly suppressed DAB2 expression at both protein and mRNA levels in NPCs (Figure S11A, B), suggesting that FTH1 contributes to the recovery of endogenous DAB2. However, given that PEVs, D‑PEVs, and mFD‑PEVs all upregulated DAB2 in NPCs to varying degrees, the restoration of endogenous DAB2 by mFD‑PEVs under oxidative stress may not rely solely on FTH1 regulation but may also involve PEVs‑associated components and amelioration of cellular homeostasis. In summary, mFD‐PEVs partially promote the recovery of endogenous DAB2 expression, which may be partly dependent on FTH1 delivery and also associated with PEV‐derived components and improved cellular homeostasis. At the same time, it restores the the SLC7A11/GPX4 antioxidant defense system, stabilize mitochondrial homeostasis and intracellular redox balance, and ultimately inhibit oxidative stress and ferroptosis in NPCs.

### 2.6 mFD‐PEVs Reversed IDD in Rats

**FIGURE 6:**
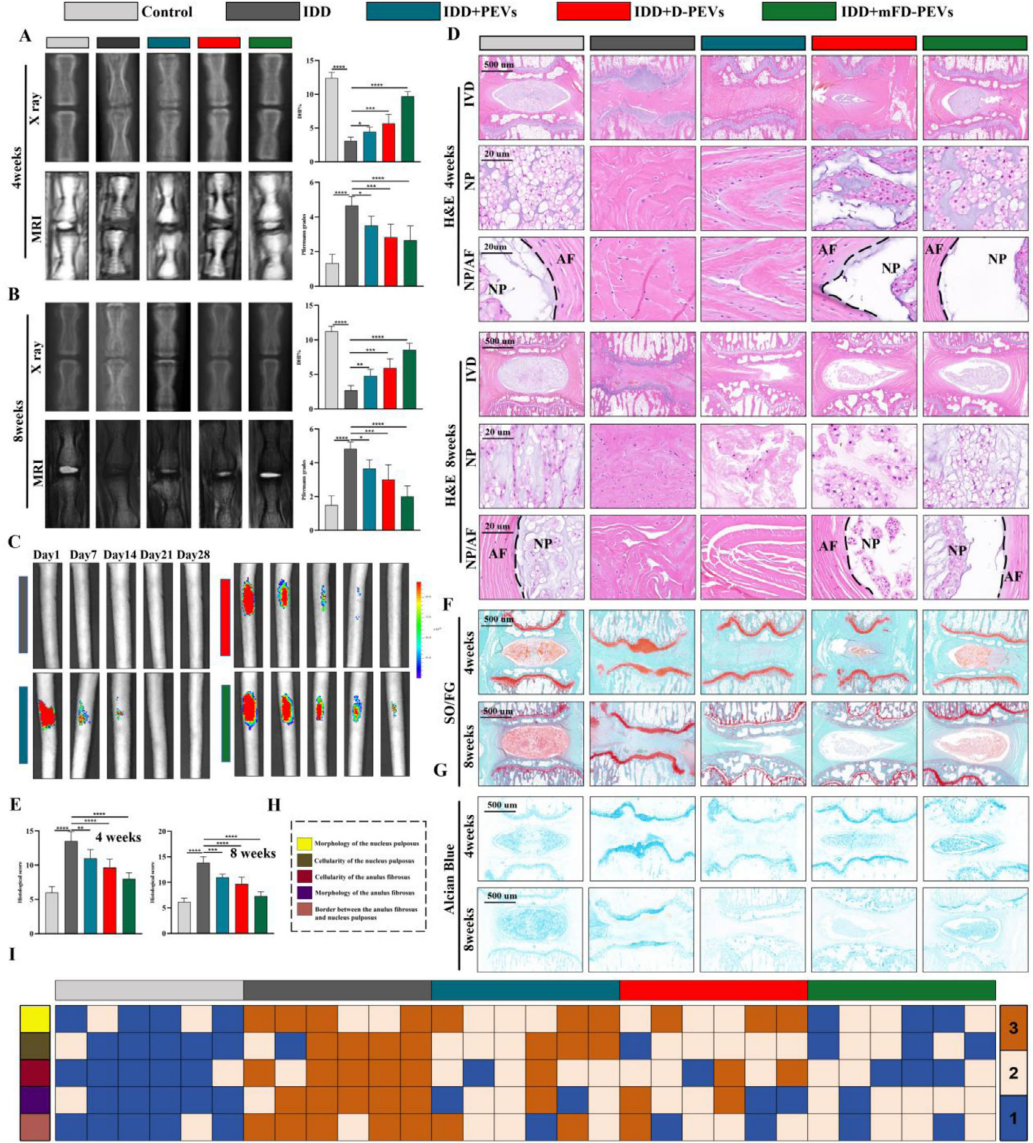
mFD‐PEVs reversing IDD in rats. (A, B) Imaging examinations of intervertebral discs by x‐ray and MRI at 4 and 8 weeks. *n* = 6, ^*^ *p* < 0.01; ^**^ *p* < 0.01; ^***^ *p* < 0.001; ^****^ *p* < 0.0001. (C) In vivo imaging reveals the retention time of PEVs in intervertebral discs. *n* = 6. (D, E) HE staining was used to evaluate the morphology and histological score of the NP at 4 and 8 weeks. *n* = 6, ^**^ *p* < 0.01; ^***^ *p* < 0.001; ^****^ *p* < 0.0001. (F) Safranin O‐Fast Green was used to evaluate the morphology of the NP at 4 and 8 weeks. *n* = 6. (G) Evaluation of collagen content by alcian blue staining at 4 and 8 weeks. *n* = 6. (H, I) Quantitative analysis of the morphology of the nucleus pulposus, cellularity of the nucleus pulposus, border between the anulus fibrosus and nucleus pulposus, morphology of the anulus fibrosus, and cellularity of the anulus fibrosus. *n* = 6. Data are expressed as mean ± standard deviation. Significance was determined using one‐way ANOVA.

We used the rat IDD model to evaluate the therapeutic effect after 4 and 8 weeks of mFD‐PEVs. X‐ray detection of intervertebral space height is used to assess the intervertebral disc height index (DHI) (Figure S12). DHI reflects the degree of IDD. The lower the DHI, the more severe the IDD. Compared with the control group, the IDD group showed significantly narrowed intervertebral space and decreased DHI. However, in the IDD group treated with PEVs, the effect of mFD‐PEVs in restoring intervertebral space height and DHI was the best (Figure 6A, B). Magnetic resonance imaging (MRI) was used to detect the signal intensity of the NP (Table S4). The signal intensity of the NP in the IDD group was significantly reduced, while in all treatment groups, the signal intensity of the NP in the mFD‐PEVs group was closer to that in the control group (Figure 6A,B). Cy3 dye was used to label PEVs, D‐PEVs and mFD‐PEVs and they were injected into the intervertebral space. In vivo imaging was performed to evaluate the retention of Cy3‐labeled PEVs within the intervertebral disc. The results indicated that the retention time of the intervertebral space of mFD‐PEVs was the longest (Figure 6C). We noted that the low fluorescence retention (<10%) in the PEVs group may stem from multiple causes. Unlike well‐vascularized tissues, the degenerated disc is an avascular, hypoxic, high‐pressure niche [51] with extensive extracellular matrix degradation [52] and elevated matrix metalloproteinase (MMP) activity [53]. These features of the degenerated disc are unfavorable for the anchoring and retention of PEVs, thereby compromising the matrix‐mediated retention of injected PEVs. Additionally, the declining fluorescence in vivo might reflect dye leakage, transfer, photobleaching, or quenching rather than clearance of intact PEVs [54]. Critically, our DAB2‐modified mFD‐PEVs showed significantly longer retention than unmodified PEVs, strongly validating the targeting efficiency of our strategy. We next evaluated the in vivo targeting of mFD‐PEVs to NPCs. Aggrecan was used as a positive marker protein for NPCs [55]. To further determine whether NPCs represent the major in vivo target compartment of mFD‐PEVs, we performed fluorescence localization analysis using Cy3‐labeled mFD‐PEVs in stereoscopic frozen sections of rat intervertebral discs. At 1, 3, and 10 days after intradiscal injection, disc sections were stained with FITC‐labeled aggrecan to identify aggrecan‐positive NP regions. Cy3 fluorescence was predominantly localized within the aggrecan‐positive NP region. Clear spatial overlap between Cy3‐labeled mFD‐PEVs and aggrecan‐positive areas at 1 and 3 days, and Cy3 fluorescence remained detectable in the NP region at 10 days, although the signal intensity gradually decreased over time. These results suggest that NPCs as a major in vivo target compartment of mFD‐PEVs. (Figure S13). Subsequently, we evaluated the morphology of the NP. HE staining indicated that the NP in the control group remained intact and was clearly decomposed from AF, while in the IDD group, the NP disappeared and its structure was disordered, with the highest histological score, suggesting that the IDD was more severe. However, the effect of mFD‐PEVs in restoring the morphology of the NP and reducing histological scores was superior to that of other treatment groups (Figure 6D,E). Notably, after 8 weeks of treatment, the NPCs morphology exhibited improvement across all treatment groups compared to the 4 weeks’ time point, accompanied by a reduction in histological scores. Among these groups, mFD‐PEVs demonstrated the most pronounced enhancement. These findings suggest that the therapeutic efficacy of mFD‐PEVs progressively improves with extended treatment duration. It should be noted that FTH1 mRNA is expected to mediate transient expression after intracellular delivery. Therefore, the therapeutic benefit observed at 8 weeks should not be interpreted as persistent FTH1 mRNA expression throughout the entire period. The 8 week endpoint was selected as a conventional time point for evaluating radiological and histological repair in IDD models. The sustained improvement may result from early restoration of ferroptosis‐associated redox homeostasis, which interrupts the degenerative cascade involving oxidative stress, ferroptosis, mitochondrial dysfunction [56]. Safranin O‐Fast Green staining indicated that the NP structure in the IDD group was disordered compared with normal NP tissue, and the NP after mFD‐PEVs treatment was closer to that of the normal group (Figure 6F). The alcian blue staining results indicated that the contents of proteoglycan and collagen in the mFD‐PEVs group were closest to those in the normal group (Figure 6G). We performed quantitative assessments of multiple structural parameters, including the morphology of the nucleus pulposus, cellularity of the nucleus pulposus, border between the anulus fibrosus and nucleus pulposus, morphology of the anulus fibrosus, and cellularity of the anulus fibrosus. Subsequently, we visualized these quantitative data after 4 weeks of treatmen as a heatmap (Figure 6H,I). Figure S14 presents the quantitative data after 8 weeks of treatment. Furthermore, we comprehensively evaluated the in vivo biosafety of PEVs. H&E staining images of the heart, liver, spleen, lungs, and kidneys were examined, and the results showed no significant abnormalities in any of the groups (Figure S15). The above results indicate that mFD‐PEVs exhibit excellent capabilities in repairing degenerated intervertebral discs and restoring disc height.

### 2.7 mFD‐PEVs Restores the Expression of Ferroptosis Related Proteins in the NP Tissue of IDD Rats

**FIGURE 7:**
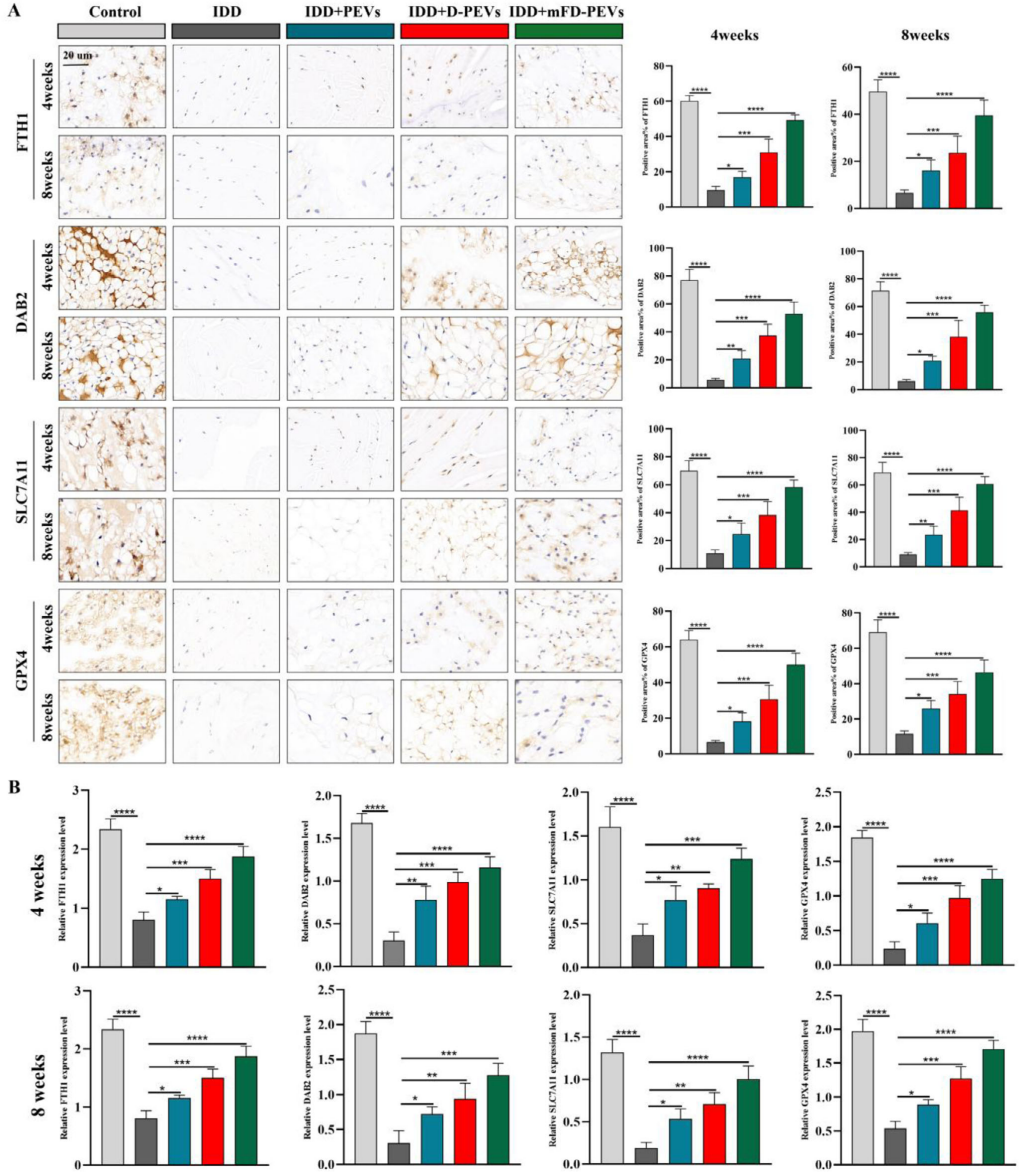
mFD‐PEVs restores the expression of related proteins in the NP of IDD rats. (A) IHC was used to detect the protein expressions of FTH1, DAB2, SLC7A11 and GPX4 in different groups at 4 and 8 weeks. *n* = 6, ^*^ *p* < 0.01; ^**^ *p* < 0.01; ^***^ *p* < 0.001; ^****^ *p* < 0.0001. (B) The mRNA levels of FTH1, DAB2, SLC7A11 and GPX4. *n* = 6, ^*^ *p* < 0.01; ^**^ *p* < 0.01; ^***^ *p* < 0.001; ^****^ *p* < 0.0001. Data are expressed as mean ± standard deviation. Significance was determined using one‐way ANOVA.

We confirmed that mFD‐PEVs restored NP morphology and alleviated IDD severity. To further explore its mechanism of action, we detected the expression levels of key regulatory proteins in the NP tissue by immunohistochemistry (IHC). The results showed that compared with the control group, the protein expressions of FTH1, DAB2, SLC7A11 and GPX4 in the IDD model group were significantly downregulated (Figure 7A). It is worth noting that among all the intervention groups, the upregulation effect of mFD‐PEVs on the above proteins was the most significant, suggesting that it may exert therapeutic effects by activating the ferroptosis defense pathway. The RT‐PCR results indicated that mFD‐PEV significantly restored the mRNA levels of FTH1, DAB2, SLC7A11 and GPX4 (Figure 7B). This further confirms the excellent therapeutic effect of mFD‐PEV on IDD rats.

### 2.8 Transcriptomic Analysis Reveals that mFD‐PEVs Restore Redox Defense and Suppress Ferroptosis‐Associated Signatures

**FIGURE 8:**
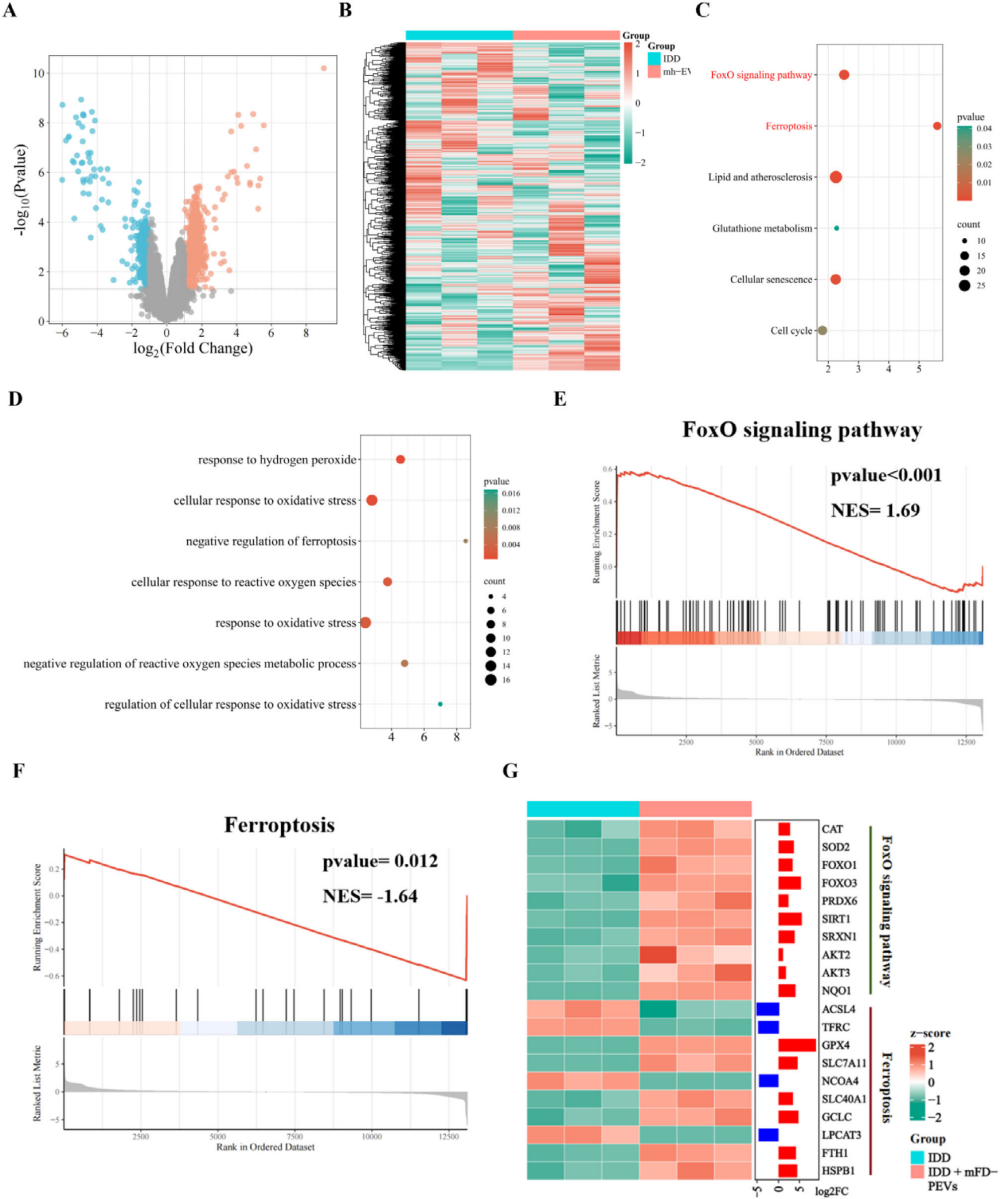
Transcriptome analysis of IDD in rats treated with mFD‐PEVs. (A, B) Volcano maps and heat map analysis of differential genes. (C) KEGG analysis of the pathways for enrichment of DEGs. (D) GO analysis of DEGs. (E) GSEA results. (F) String plots reveal the relationship between genes and pathways. (G) The heat map shows the differential expression of some genes in the IDD and mFD‐PEVs groups.

To gain a deeper understanding of the molecular mechanism of mFD‐PEVs in the treatment of IDD, we conducted mRNA transcriptome sequencing of the NP in the IDD and the mFD‐PEVs group. The results identified 670 upregulated and 475 downregulated genes. All differentially expressed genes (DEGs) were visualized using volcano plots and heat maps (Figure 8A, B). Kyoto Encyclopedia of Genes and Genomes (KEGG) analysis revealed that the FoxO signaling pathway, ferroptosis, and glutathione metabolism were significantly enriched (Figure 8C). Moreover, these pathways are involved in cellular oxidative stress. Subsequently, Gene Ontology (GO) enrichment analysis was performed to assess gene functions. The results of the Biological process (BP) indicated that DEGs are enriched in response to hydrogen peroxide, cellular response to oxidative stress, and negative regulation of ferroptosis, and these pathways are related to cellular oxidative stress (Figure 8D). The results of gene set enrichment analysis (GSEA) indicated that the mFD‐PEVs group promoted the upregulation of the FoxO signaling pathway (Figure 8E). Conversely, Ferroptosis pathway was downregulated (Figure 8F). The heat map results indicated that mFD‐PEVs significantly promoted the expression of antioxidant genes such as CAT, SOD2 and FOXO1, and inhibited the expression of ferroptosis driving genes such as ACSL4, TFRC and LPCAT3 (Figure 8G). These results indicate that mFD‐PEVs may exert therapeutic effects in IDD by activating antioxidant defense mechanisms in NPCs.

From a translational perspective, although mFD‐PEVs showed promising efficacy in this preclinical study, several translational issues remain to be addressed. Scalable and standardized mFD‐PEVs manufacturing, reproducible donor cell culture, efficient purification, and rigorous quality control will be required for future application. Future studies should establish reproducible cell culture conditions and rigorous quality control criteria. Batch variability should be monitored by assessing particle size distribution, particle concentration, PEV marker expression, DAB2 incorporation, FTH1 mRNA loading efficiency. In addition, the efficiency and stability of FTH1 mRNA loading, long‐term storage conditions, and biosafety should be systematically optimized. Therefore, further studies are needed to support future clinical translation of mFD‐PEVs for IDD treatment.

## 3 Conclusion

In this study, we developed dual‐engineered NPPCs‐derived extracellular vesicles, termed mFD‐PEVs. DAB2 modification enhanced the interaction and uptake of mFD‐PEVs by NPCs, whereas FTH1 mRNA delivery contributed to the restoration of iron/redox homeostasis under oxidative stress. Mechanistically, mFD‐PEVs reduced Fe^2+^ accumulation, ROS production, lipid peroxidation, and mitochondrial dysfunction, while preserving the SLC7A11/GPX4‐associated antioxidant defense axis and attenuating ferroptosis‐associated injury in NPCs. In vivo, mFD‐PEVs effectively alleviated IDD, improved disc structural preservation, and restored ferroptosis‐related protective markers in degenerative NP tissue. Transcriptomic analysis further indicated that mFD‐PEVs mainly modulated oxidative stress, redox homeostasi, and ferroptosis‐related signatures. In summary, this study proposes a strategy for genetically modifying PEVs to modulate iron/redox homeostasis and inhibit NPC damage associated with ferroptosis, providing a potential therapeutic approach for delaying the progression of IDD.

## 4 Experimental Section

### 4.1 Collection of Human Intervertebral Disc Samples

This study was approved by the Ethics Committee of Huai'an Hospital Affiliated to Xuzhou Medical University (Approval Number: HEYLL2022689). All patients provided written informed consent prior to the procedure. We used the Pfirrmann grading system to classify the NP tissues from the patients into the normal group (grades I‐III) and the degenerative group (grades III‐V). Patients with Pfirrmann MRI grades I‐II were diagnosed with new thoracolumbar fractures and scoliosis, while those with grades III‐V were diagnosed with lumbar intervertebral disc protrusion, lumbar spinal stenosis, or lumbar spondylolisthesis [57]. We documented the clinical and pathological characteristics of all patients (Table S1).

### 4.2 Animal Model

Male SD rats (250–300 g) were obtained from the Laboratory Animal Center of Xuzhou Medical University (Xuzhou, China), and all procedures were approved by the Laboratory Animal Ethics Committee of Xuzhou Medical University (Approval No. 202411T008). SD rats were randomly assigned to groups (*n* = 6 per group). Under x‐ray guidance, a 22‐gauge needle was inserted into the Co7/Co8 intervertebral disc to a depth of 3 mm, rotated 360°, and held for 30 s to induce disc degeneration. Thereafter, 5 µL of PEVs (1 µg/µL) were injected into the disc center via a microinjector every 7 days.

### 4.3 Statistical Analysis

Prior to statistical analysis, the raw data were examined to eliminate data entry errors and technical malfunctions. For measured values relative to the control group, the data were normalized to the mean of the control group. All quantitative results were derived from at least three independent biological replicates, and the specific sample size (n) for each experiment is indicated in the corresponding figure legend. All data were presented as mean ± standard deviation. Statistical analyses were performed using the Prism 9 software (GraphPad Software, La Jolla, CA, USA). To assess group differences, the data were subjected to statistical analysis using a two‐tailed Student's *t*‐test or one‐way analysis of variance, followed by a post‐hoc Tukey's multiple comparison test. Statistical significance was set at ^*^ *p* < 0.05, ^**^ *p* < 0.01, ^***^ *p* < 0.001, and ^****^ *p* < 0.0001. “ns” denotes non‐significant differences.

## Author Contributions

J. Yan, Y. Lin, S. Miao and M. Chao contributed equally to this research work.

## Conflicts of Interest

The authors declare no conflicts of interest.

## Supporting information




**Supporting File**: advs77329‐sup‐0001‐SuppMat.docx.

## Data Availability

The data that support the findings of this study are available from the corresponding author upon reasonable request.
