# Supplementary material for: Dual‐Gene Edited Extracellular Vesicles Remodel the Redox Homeostasis to Inhibit Ferroptosis in Intervertebral Disc Degeneration
Source: Adv Sci (Weinh). 2026 Aug 20:e77329. Online ahead of print. doi: 10.1002/advs.77329 (PMC13491356; doi:10.1002/advs.77329)
Supplement: Supplementary file 1 [file ADVS-9999-e77329-s001.docx]

Supporting Information

**Dual-Gene Edited Extracellular Vesicles Remodel the Redox Homeostasis to Inhibit Ferroptosis in Intervertebral Disc Degeneration**

Jing Yan, Yazhou Lin, Shuo Miao, Minghao Chao, Chengbai Dai, Ziwen Hao, Rui Chen, Ming Guan*, Bin Pan*, Fenglei Gao* and Quan Zhou*,

J. Yan, S. Miao, Z. Hao, R. Chen, Q. Zhou.

Department of Orthopedics;

The Affiliated Huai'an Hospital of Xuzhou Medical University;

Huaian 223000, China.

Email: wuque1@126.com (Q. Zhou).

B. Pan, F. Gao.

Department of Spine Surgery;

The Affiliated Hospital of Xuzhou Medical University;

Jiangsu 221004, China.

Email: [panbinxyfygk@163.com](mailto:panbinxyfygk@163.com) (B. Pan), flgao@xzhmu.edu.cn (F. Gao).

C. Dai, F. Gao.

Key Laboratory of New Drug Research and Clinical Pharmacy of Jiangsu Province;

Xuzhou Medical University;

Jiangsu 221004, China.

M. Chao.

Department of Orthopaedic Surgery,

Lishui Central Hospital and Fifth Affiliated Hospital of Wenzhou Medical University, Zhejiang 323000, China.

Y. Lin

Department of Orthopedics,

Ruijin Hospital,

Shanghai Jiaotong University School of Medicine,

200020, China.

M. Guan

Department of Laboratory Medicine,

Huashan Hospital, Shanghai Medical College,

Fudan University,

Shanghai 200040, China.

Email: [guanming88@yahoo.com](mailto:guanming88@yahoo.com) (M. Guan)

**Materials and Methods**

**Instruments:** Separation of EVs by ultracentrifugation (Optima XPN-100, Beckman Coulter, USA). The morphology of the samples was observed under the TecnaiTM G2 TWIN (FEI, USA) transmission electron microscope. Fluorescence images were obtained on a fluorescence inverted microscope (IX73P1F, Olympus, Japan) and a confocal laser scanning microscope (Leica STELLARIS 5, Germany). Perform flow cytometry analysis on the FACS Canto II flow cytometry (Becton Dickinson, USA). The IVIS imaging system (Perkin Elmer Lumina S5, USA) is used for in vivo fluorescence imaging.

**Transcriptome data:** The mRNA data set from human degenerated and normal intervertebral disc tissues (GSE167199) was downloaded from the GEO database. This data set contains 3 normal and 3 degenerated intervertebral disc tissues.

**Identification of differentially expressed genes:** The limma package was used to screen for differentially expressed genes between normal and degenerated intervertebral disc tissues. |fold change (FC)| > 1 and p < 0.05 were defined as differentially expressed genes. ggplot2 and pheatmap were used to create volcano plots and heatmaps for visual analysis of the differentially expressed genes^[1]^.

**Functional enrichment analysis:** The clusterProfiler was used to perform Gene Ontology (GO) and Kyoto Encyclopedia of Genes and Genomes (KEGG) analyses on the differentially expressed genes^[1]^.

**Isolation of nucleus pulposus progenitor cells (NPPCs) from newborn rats:** The nucleus pulposus (NP) tissue of newborn rats was digested with collagenase II (0.1-0.2%) in DMEM/F12 medium for 30 minutes. Then, it was filtered through a 70-micron cell filter and washed three times with PBS. Subsequently, the NPPCs were resuspended and cultured in DMEM/F12 medium (10% FBS, 1% penicillin/streptomycin)^[2]^.

**Isolation of nucleus pulposus cells (NPCs):** The NP tissue was cut into 2-3 mm³ slices under aseptic conditions, washed with PBS three times, and digested with 0.25% trypsin at 37°C for 0.5 hours, or with 0.2% type II collagenase (Solarbio, Beijing, China) for 3 hours, and then cultured in DMEM/F12 medium. The medium was changed every 3 days. The cells were incubated at 37°C and 5% CO₂^[3]^.

**Extraction of extracellular vesicles (PEVs) from NPPCs:** The experiment was conducted according to the instructions of the BeyoExo™ Enhanced Exosome Isolation Kit from Cell Culture Media by Precipitation Method (Beyotime, Guangzhou, China). The kit was used according to the manufacturer’s protocol to remove residual soluble proteins and non-vesicular contaminants. First, centrifuge the supernatant of NPPCs at 500g for 5 minutes to remove the suspended cells. Then, centrifuge again at 10000g for 30 minutes to remove dead cells, then the supernatant was filtered through a 0.22 µm needle filter to further remove larger cell vesicles and apoptotic bodies and other impurities. Subsequently, BeyoExo™ enhanced EVs extraction reagent for cells was added. Afterwards, centrifuge at 12,000g for 2 minutes to rremove residual soluble proteins and non-vesicular contaminants. Subsequently, The impurities were removed by resuspending and briefly centrifuging. The precipitate was resuspended with an appropriate amount of PBS. Then, it was centrifuged at 12,000g for 2 minutes at 4ºC, and the supernatant was collected. If the precipitate was abundant, the supernatant could be centrifuged multiple times at 12,000g for 2 minutes at 4ºC until no obvious precipitate remained.Finally, re-seal the PEVs in 200 microliters of PBS for subsequent analysis.

**Loading of FTH1:** The overexpressed FTH1 mRNA used in this experiment was synthesized by Shanghai Sangon Biotech. We prepared D-PEVs expressing FTH1 using electroporation technology^[4]^. First, D-PEVs were resuspended in electroporation buffer, then transferred to the electroporation cup, and 10 ug of D-PEVs were added with 5 ug of FTH1 mRNA, resulting in a final volume of 50 ul. The electroporation cup reaction conditions were set at 400 volts, 125 micro-watts, and two pulses were performed. Then the PEVs were transferred to a 1.5 ml tube. EDTA solution at 1 mmol/L was added to reduce the aggregation of plasmid DNA. The PEVs suspension was then transferred to an ultrafiltration tube and centrifuged at 5000 r/min for 10 minutes at 4 °C to remove excess buffer and unincorporated FTH1mRNA.

**Determination of PEVs uptake:** PEVs were labeled according to the instructions of the PKH26 staining kit (Beyotime, Guangzhou, China), and then co-cultured with nucleus cells for 6 h. Finally, the uptake of exosomes by nucleus cells was detected by immunofluorescence and flow cytometry. Chlorpromazine (Beyotime, Guangzhou, China)，Dynasore (Beyotime, Guangzhou, China)， Filipin (Beyotime, Guangzhou, China) and Wortmannin (Beyotime, Guangzhou, China) are used to verify the endocytosis mechanism of mFD-PEVs.

**Lysosome escape:** Assess the lysosome escape situation of PEVs. NPCs were seeded into 24-well plates (3×10^5^ cells per well) and incubated for 12 hours. PKH26-labeled PEVs were added to the FBS-free DMEM/F12 culture medium, and the cells were cultured for 1, 2, 4, and 6 hours. The cells were washed three times with PBS and stained with Lysotracker (Thermo Scientific, USA) for 15 minutes, and stained with DAPI for 5 minutes to visualize the cell nuclei. Finally, images were captured using a confocal laser scanning microscope (Leica STELLARIS 5, Germany).

**siRNA-mediated FTH1 knockdown in NPCs:** The si-FTH1 used in this experiment was synthesized by Shanghai Sangon Biotech. NPCs were seeded into culture plates and transfected with si-FTH1 or negative control siRNA using a transfection reagent according to the manufacturer’s instructions. After transfection for 24h, the protein and mRNA levels of FTH1 and DAB2 by WB and RT-qPCR.

**Proteinase K protection assay for determining DAB2 localization on mFD-PEVs:** Briefly, equal numbers of mFD-PEVs were divided into three groups: untreated mFD-PEVs, proteinase K-treated mFD-PEVs, and proteinase K+Triton X-100-treated mFD-PEVs. For the proteinase K-treated group, mFD-PEVs were incubated with proteinase K at 37°C for 30 min to digest proteins exposed on the outer surface of intact vesicles. For membrane permeabilization, mFD-PEVs were pretreated with Triton X-100 and then incubated with proteinase K under the same conditions. The digestion reaction was terminated by adding protease inhibitor or PMSF. After treatment, EV proteins were extracted and subjected to western blot analysis.

**RNase protection assay for determining FTH1 mRNA encapsulation in mFD-PEVs:** Equal numbers of mFD-PEVs were divided into three groups: untreated mFD-PEVs, RNase-treated mFD-PEVs, and Triton X-100+RNase-treated mFD-PEVs. In the RNase-treated group, intact mFD-PEVs were incubated with RNase A at 37°C for 30 min to degrade externally exposed RNA. In the Triton X-100 plus RNase-treated group, mFD-PEVs were first permeabilized with Triton X-100 to disrupt the vesicle membrane, followed by RNase A treatment under the same conditions. After treatment, total RNA was extracted from mFD-PEVs using an RNA extraction reagent according to the manufacturer’s instructions. FTH1 mRNA levels were then quantified by RT-qPCR.

**Imaging analysis and histological examination:** X-ray and MRI imaging examinations were conducted 4 and 8 weeks after treatment. The existing intervertebral disc height index (DHI) and Pfirrmann grading criteria^[5]^ were adopted. After the MRI examination, the rats were killed, and the intervertebral disc specimens were fixed, decalcified, dried, embedded in paraffin, and then sectioned into 4 um thick slices. The slices were stained with H&E, Safranin O/fast green, and Alcian Blue, and the degree of intervertebral disc degeneration was evaluated^[5]^. The criteria for determining the degree of intervertebral disc degeneration are shown in **Table 5**. At the same time, the expressions of proteins such as FTH1, DAB2, GPX4, SLC7A11, FOXO3, BCL2 and MMP3 in the NP tissue were verified.

**Intracellular fluorescence imaging:** Different PEVs labeled with CY3 were injected into the intervertebral spaces of rats, and then the distribution of CY3 fluorescence signals over time was monitored using an in vivo imaging system (PerkinElmer Lumina S5 from the United States).

**Cellular immunofluorescence:** First, the NPCs were washed with PBS, then fixed with 4% formaldehyde, permeabilized with 0.5% Triton X-100, and blocked with 5% BSA. Next, an antibody was added and cultured at 4°C overnight, and then FITC or Cy3-labeled fluorescent secondary antibody was added at room temperature in the dark. DAPI (Beyotime, Guangzhou, China) was used for cell nucleus staining. Finally, images were captured using a confocal laser scanning microscope (Leica STELLARIS 5 from Germany).

**Flow cytometry detection:** Collect the NPCs and conduct the cell viability detection according to the instructions of the kit (Beyotime, Guangzhou, China). We analyzed the cells using a flow cytometer (Beckman Coulter, California, USA).

**RT-qPCR:** RNA was extracted using Trizol (TianGen, Beijing, China), and RNA expression was quantified using LightCycler480II (Roche Diagnostics, Indianapolis, USA). A 20 μl PCR reaction system was prepared, with 40 cycles of 30 seconds at 95 °C, 10 seconds at 95 °C, and 30 seconds at 60 °C. The primer sequences are listed in **Table 2**.

**Western Blot (WB):** Protein extraction was performed using RIPA and PMSF (Beyotime, Guangzhou, China), and protein concentration was determined using the BCA kit (Beyotime, Guangzhou, China). The following rabbit anti-FTH1 (11682-1-AP, 1:1000, Proteintech, China), rabbit anti-DAB2 (10109-2-AP, 1:1000, Proteintech, China), rabbit anti-GPX4 (1:2000, 30388-1-AP, Proteintech, China), rabbit anti-SLC7A11 (1:3000, 26864-1-AP, Proteintech, China), and mouse anti-GAPDH (1:50,000, 60004-1-Ig, Proteintech, China) were used. Goat anti-rabbit or goat anti-mouse antibodies were sourced from (1:50,000, V926-32211/32210, Vicmed, China). GAPDH was used as the control. The sources of the antibodies used in the experiment are shown in **Table 3**.

**Mitochondrial analysis:** The JC-1 reagent (Beyotime, Guangzhou, China) was used to detect changes in mitochondrial membrane potential. The MitoSox reagent (Beyotime, Guangzhou, China) was used to detect changes in intramitochondrial superoxide. The MitoTracker Red reagent (Invitrogen, USA) was used to visualize mitochondrial morphology and analyze changes in mitochondrial length^[6, 7]^. All of them were captured under a confocal laser scanning microscope (Leica STELLARIS 5, Germany). For each group, NPC was collected and mitochondrial morphology was examined under a transmission electron microscope (TEM).

**Detection of MDA, GSH, ROS and iron content:** MDA and GSH levels were measured using respective kits (Beyotime, Guangzhou, China) at 532 nm and 412 nm, respectively. ROS levels were analyzed using the ROS detection kit (Beyotime, Guangzhou, China) in a flow cytometer (Beckman Coulter, California, USA). Iron content was determined according to the kit instructions (Beyotime, Guangzhou, China). The optical density values were measured at 520 nm using a microplate reader (Thermo Fisher Scientific, USA).

**Lipid Peroxidation Assay:**The lipid peroxidation level of the NPCs was detected using the Lipid Peroxidation Assay Kit with BDPY 581/591 C11 reagent (Beyotime, Guangzhou, China).

**Table 1.Clinical characteristics of surgery patients**

| Samples | Type | Gender | Age(years) | Diagnosis | Tissues | Pfirrman grade |
| --- | --- | --- | --- | --- | --- | --- |

| Sample 1 | Normal | Male | 51 | Thoracolumbar fracture | NP | II |
| --- | --- | --- | --- | --- | --- | --- |
| Sample 2 | Normal | Female | 58 | Thoracolumbar fracture | NP | I |
| Sample 3 | Normal | Male | 45 | Thoracolumbar fracture | NP | II |
| Sample 4 | Normal | Male | 42 | Thoracolumbar fracture | NP | II |
| Sample 5 | Normal | Female | 67 | Lumbar disc herniation | NP | II |
| Sample 6 | Normal | Male | 33 | Lumbar disc herniation | NP | I |
| Sample 7 | Normal | Female | 31 | Scoliosis | NP | I |
| Sample 8 | Normal | Male | 19 | Scoliosis | NP | II |
| Sample 9 | Normal | Female | 23 | Scoliosis | NP | II |
| Sample 10 | IDD | Female | 17 | Lumbar spinal stenosis | NP | I |
| Sample 11 | IDD | Male | 45 | Lumbar spinal stenosis | NP | V |
| Sample 12 | IDD | Female | 40 | Lumbar spinal stenosis | NP | IV |
| Sample 13 | IDD | Female | 53 | Lumbar spinal stenosis | NP | IV |
| Sample 14 | IDD | Male | 67 | Lumbar spinal stenosis | NP | IV |
| Sample 15 | IDD | Female | 32 | Lumbar disc herniation | NP | IV |
| Sample 16 | IDD | Male | 46 | Lumbar disc herniation | NP | IV |
| Sample 17 | IDD | Male | 30 | Lumbar disc herniation | NP | IV |
| Sample 18 | IDD | Male | 20 | Lumbar disc herniation | NP | V |
| Sample 19 | IDD | Female | 48 | Lumbar disc herniation | NP | IV |
| Sample 20 | IDD | Female | 52 | Lumbar disc herniation | NP | V |

**Table 2. Primer Sequence**

| **FTH1** | GCTTCAACAGTGCTTGGACG | TGAAGGAAGATTCGGCCACC |
| --- | --- | --- |
| **DAB2** | CAAACTGAAATCGGAAAGCAAAGA | GGGCAGGAGCACTTGTTTTG |
| **SLC7A11** | GCCCAAGGGGAGACACAAAA | TTGAAGGTTCTTTGCCGTGC |
| **GPX4** | TCACCAAGTTTGGACACCGT | ATAGTGGGGCAGGTCCTTCT |
| **GAPDH** | GCTCATTTGCAGGGGGGAG | GTTGGTGGTGCAGGAGGCA |
| **FOXO3**  **FOXO1**  **BAX** | GCCCGGGATAACCAACTCTC  CAGCCGCCACATTCAACAGG  TTCATCCAGGATCGAGCAGG | TGTGCCGGATGGAGTTCTTC  GCTCTTGACCATCCACTCGT  TGTCCAGCCCATGATGGTTC |
| **BCL2** | AAAAATACAACATCACAGAGGAAGT | TCCCGGTTATCGTACCCTGT |
| **MMP3**  **MMP9** | TGAGGACACCAGCATGAACC  TCTATGGTCCTCGCCCTGAA | ACTTCGGGATGCCAGGAAAG  TTGTATCCGGCAAACTGGCT |

**Table 3. Antibodies used in this study**

| **Company** | **Antibodies** | **Catalog no.** |
| --- | --- | --- |
| Proteintech | Anti-FTH1 | 11682-1-AP |
| Proteintech | Anti-DAB2 | 10109-2-AP |
| Proteintech | Anti-SLC7A11 | 26864-1-AP |
| Proteintech | Anti-GPX4 | 30388-1-AP |
| Proteintech | Anti-CD73 | 12231-1-AP |
| Proteintech | Anti-CD90 | 66766-1-Ig |
| Invitrogen | Anti-FOXO3 | PA5-116792 |
| Proteintech | Anti-BCL2 | 60178-1-Ig |
| Abcam | Anti-MMP3 | ab52915 |
| Proteintech | Anti-FOXO1 | 18592-1-AP |
| Proteintech | Anti-Bax | 50599-2-Ig |
| Proteintech | Anti-MMP9 | 10375-2-AP |

**Table 4. Pfirrmann classification**

| **Grade** | **Structure** | **Distinction of Nucleus and Anulus** | **Signal Intensity** | **Height of Intervertebral Disc** |
| --- | --- | --- | --- | --- |
| **I** | **Homogeneous** | **Clear** | **Hyperintense, isointense to cerebrospinal fluid** | **Normal** |
| **Ⅱ** | **Inhomogeneous**  **with or without horizontalbands** | **Clear** | **Hyperintense, isointense to cerebrospinal fluid** | **Normal** |
| **Ⅲ** | **Inhomogeneous, gray** | **Unclear** | **Intermediate** | **Normal to slightly decreased** |
| **IV** | **Inhomogeneous, gray to black** | **Lost** | **Intermediate to hypointense** | **Normal to moderately decreased** |
| **V** | **Inhomogeneous, black** | **Lost** | **Hypointense** | **Collapsed disc space** |

**Table 5. Histological Grading Scale**

I. **Cellularity of the anulus fibrosus Grade:**

1.Fibroblasts comprise more than 75%of the cells

2.Neither fibroblasts nor chondrocytes comprise more than 75%of the cells

3.Chondrocytes comprise more than 75%of the cells

**II. Morphology of the anulus fibrosus Grade:**

1.Well-organized collagen lamellae without ruptured or serpentine fibers

2.Inward bulging,ruptured or serpentine fibers in less than one third of the annulus

3.Inward bulging,ruptured or serpentine fibers in more than one third of the annulus

**III. Border between the anulus fibrosus and nucleus pulposus**

Grade:

1.Normal,without any interruption

2.Minimal interruption

3.Moderate or severe interruption

**IV. Cellularity of the nucleus pulposus Grade:**

1.Normal cellularity with stellar shaped nuclear cells evenly distributed throughout the nucleus

2.Slight decrease in the no.of cells with some clustering

3.Moderate or severe decrease(>50%)in the number of cells with all the remaining cells clustered and separated by dense areas of

proteoglycans

**V. Morphology of the nucleus pulposus Grade:**

1.Round,comprising at least half of the disc area in midsagittal sections

2.Rounded or irregularly shaped,comprising one quarter to half of the disc area in midsagittal sections

3.Irregularly shaped,comprising less than one quarter of the disc area in midsagittal sections

**Figure S1**. **FTH1 and DAB2 are the key differentiating genes.** (A) Volcano plot for screening differentially expressed genes. (B) Heatmap for visualizing the differences in genes of the ferroptosis pathway, with FTH1 expression downregulated. (C) Heatmap for visualizing the differences in genes of the endocytosis pathway, with DAB2 expression downregulated. (D) Protein interaction indicates that FTH1 and DAB2 are the hub genes for all the downregulated genes in the ferroptosis and endocytosis pathways, respectively. (E) FTH1 is involved in the ferroptosis pathway. (F) DAB2 is involved in the endocytosis pathway.

**Figure S2.** **TBHP induces ferroptosis in NPCs.** (A) Effect of different concentrations of TBHP on cellular lipid peroxidation levels. n = 3. ( (B) Effect of different concentrations of TBHP on cell proliferation. n = 3, ***p < 0.001; ****p < 0.0001. (C) Fer-1 reverses TBHP-induced downregulation of SLC7A11 and GPX4 protein expression. n = 3, **p < 0.01; ***p < 0.001; ****p < 0.0001. (D, E) Fer-1 reverses TBHP-induced downregulation of SLC7A11 and GPX4 gene expression. n = 3, **p < 0.01; ***p < 0.001; ****p < 0.0001. (F) MDA content. n = 3, **p < 0.01; ***p < 0.001. (G) Iron ion content. n = 3, ***p < 0.001; ****p < 0.0001. (H) GSH content. n = 3, ***p < 0.001; ****p < 0.0001. (I) Fer-1 attenuates lipid peroxidation levels in the TBHP-induced group. n = 3, ****p < 0.0001. (J) Fer-1 inhibits ROS levels in the TBHP-induced group. n = 3, ***p < 0.001; ****p < 0.0001. (K) Fer-1 restores cell viability in the TBHP-induced group. n = 3, ****p < 0.0001. Data are expressed as mean ± standard deviation. Significance was determined using one-way ANOVA.

**Figure S3. TBHP-induced NPCs model at least recapitulates the core redox-related molecular features of clinical IDD.** (A) The protein and gene expression of SLC7A11 and GPX4. n = 3, **p < 0.01; ***p < 0.001. (B, C) The protein and gene expression of FTH1, DAB2, SLC7A11 and GPX4. n = 3, **p < 0.01; ***p < 0.001; ****p < 0.0001. (D) Summary heatmap of the gene expression profiles for FTH1, DAB2, SLC7A11, and GPX4. n = 3. Data are expressed as mean ± standard deviation. Significance was determined using a two-tailed Student's t-test.

**Figure S4**. **NPCs have a stronger affinity for PEVs.** (A) Extraction of exosomes derived from mesenchymal stem cells. (B) Characterization of MSCs-EVs. n = 3. (C, D) Differences in the uptake of MSCs-EVs and NPPCs-EVs by nucleus cells. n = 3.

**Figure S5. Characterization of mFD-PEVs.** (A) DAB2 incorporation. n = 3, **p < 0.01; ns, not significant. (B, C) The expression of DAB2 protein in mFD-PEVs was detected by treating with protease K and Triton X-100. n = 3, ****p < 0.0001; ns, not significant. (D) Restriction enzyme-cut FTH1 plasmid. (E) Loading efficiency of FTH1 in mFD-PEVs. n = 3, ****p < 0.0001. Data are expressed as mean ± standard deviation. The data were subjected to statistical analysis using a two-tailed Student's t-test or one-way analysis of variance, followed by a post-hoc Tukey's multiple comparison test.

**Figure S6. The endocytic mechanism of mFD-PEVs and its safety for NPCs.** (A) NPCs were cultured under different inhibition conditions. Representative confocal fluorescence micrographs showed the internalization of PKH26-labeled (red) PEVs in these groups. Chlorpromazine: clathrin-mediated endocytosis inhibitor; Dynasore: dynamin inhibitor; Filipin: caveolae-dependent endocytosis inhibitor; Wortmannin: inhibitor of the phosphoinositide 3-kinase. n = 3. (B) Fluorescence intensity of PKH26-labeled PEVs taken up by NPCs under different inhibition conditions by flow cytometry. n = 3. (C) Live/dead cell staining. n = 3.

**Figure S7. mFD-PEVs successfully deliver FTH1 mRNA into NPCs induced by TBHP and support its subsequent translation.** (A) NPCs uptake of mFD-PEVs increased progressively over time, exhibiting a time-dependent manner. n = 3. (B) mRNA levels of FTH1 were elevated to varying degrees. n = 3, *p < 0.05; ***p < 0.001; ****p < 0.0001. (C, D) Protein levels of FTH1 were also elevated to varying degrees. n = 3, *p < 0.05; ***p < 0.001; ****p < 0.0001. (E) Cellular immunofluorescence experiment was used to detect the protein expression of FTH1 at different times. n = 3. Data are expressed as mean ± standard deviation. Significance was determined using one-way ANOVA.

**Figure S8.** **mFD-PEVs directly regulate the SLC7A11/GPX4 pathway.** (A) The protein expression of SLC7A11 and GPX4. n = 3, **p < 0.01; ***p < 0.001. (B) The gene expression of SLC7A11 and GPX4. n = 3, **p < 0.01; ****p < 0.0001. (C) Cellular immunofluorescence was used to detect the expression of SLC7A11 and GPX4. n = 3. Data are expressed as mean ± standard deviation. Significance was determined using one-way ANOVA.

**Figure S9.** **FTH1 serves as a critical functional component of the mFD-PEVs mediated protection.** (A, B) Protein and mRNA expressions of FTH1, DAB2, SL7A11 and GPX4. n = 3, **p < 0.01; ***p < 0.001; ****p < 0.0001; ns, not significant. (C) GSH content. n = 3, ***p < 0.001; ****p < 0.0001; ns, not significant. (D) Fe^2+^ content. n = 3, ****p < 0.0001; ns, not significant. (E) MDA content. n = 3, ****p < 0.0001; ns, not significant. (F) ROS levels. n = 3. (G) lipid peroxidation levels. n = 3. Data are expressed as mean ± standard deviation. Significance was assessed using one-way ANOVA followed by Tukey's multiple-comparison test.

**Figure S10.** **mFD-PEVs promotes endogenous DAB2 expression.** (A, B) The protein expressions of DAB2. n = 3, **p < 0.01; ***p < 0.001; ****p < 0.0001. (C) The mRNA expressions of DAB2. n = 3, *p < 0.05; ***p < 0.001; ****p < 0.0001. Data are expressed as mean ± standard deviation. Significance was determined using one-way ANOVA.

**Figure S11.** **FTH1 regulates the expression of DAB2 in NPCs.** (A) The protein expressions of DAB2. n = 3, **p < 0.01; ns, not significant.(B) The mRNA expressions of DAB2. n = 3, ***p < 0.001; ns, not significant. Data are expressed as mean ± standard deviation. Significance was determined using one-way ANOVA.

**Figure S12. Measurement of Disc Height Index (DHI) for Intervertebral Space Height in X-ray Images.**

**Figure S13. In vivo localization of Cy3-labeled mFD-PEVs in aggrecan positive NP regions (n = 3).**

**Figure S14. The quantitative data after 8 weeks of treatment (n = 6).**

**Figure S15. H&E staining images of major organs from rats after different treatments (n = 6).**

**References**

1. K. Mu, J. Geng, Y. Dong, and W. Guo, “Identification of signature genes and relationship with immune cell infiltration in intervertebral disc degeneration,” *Frontiers in Genetics* 9 (2025): 1551124.
2. L. Liu, Y. Zhang, J. Fu., “Gli1 depletion induces oxidative stress and apoptosis of nucleus pulposus cells via Fos in intervertebral disc degeneration,” *Journal of Orthopaedic Translation* 13 (2023): 116-131.
3. Q. Xiang, J. Zhan, S. Tian., “Human iPSCs Derived MSCs-Secreted Exosomes Modulate Senescent Nucleus Pulposus Cells Induced Macrophage Polarization via Metabolic Reprogramming to Mitigate Intervertebral Disc Degeneration,” *Advanced Science* 12 (2025): e04347.
4. S. E. Andaloussi, Y. Lee, J. Li., “Exosome-mediated delivery of siRNA in vitro and in vivo,” *Nature Protocols* 7 (2012): 2112-26.
5. Y. Jin, O. Wu, Q. Chen., “Hypoxia-Preconditioned BMSC-Derived Exosomes Induce Mitophagy via the BNIP3-ANAX2 Axis to Alleviate Intervertebral Disc Degeneration,” *Advanced Science* 11 (2024): e2404275.
6. Y. Jin, O. Wu, Q. Chen., “Hypoxia-Preconditioned BMSC-Derived Exosomes Induce Mitophagy via the BNIP3-ANAX2 Axis to Alleviate Intervertebral Disc Degeneration,” *Advanced Science* 11 (2024): e2404275.
7. V. Madhu, M. M. Hernandez, P. Boneski., “The mitophagy receptor BNIP3 is critical for the regulation of metabolic homeostasis and mitochondrial function in the nucleus pulposus cells of the intervertebral disc,” *Autophagy* 19 (202): 1821-1843.
